# Major Phytochemicals: Recent Advances in Health Benefits and Extraction Method

**DOI:** 10.3390/molecules28020887

**Published:** 2023-01-16

**Authors:** Ashwani Kumar, Nirmal P, Mukul Kumar, Anina Jose, Vidisha Tomer, Emel Oz, Charalampos Proestos, Maomao Zeng, Tahra Elobeid, Sneha K, Fatih Oz

**Affiliations:** 1 Department of Postharvest Technology, College of Horticulture and Forestry, Rani Lakshmi Bai Central Agricultural University, Jhansi 284003, Uttar Pradesh, India; 2 Department of Food Technology and Nutrition, Lovely Professional University, Phagwara 144411, Punjab, India; 3 VIT School of Agricultural Innovations and Advanced Learning, Vellore Institute of Technology, Vellore 632014, Tamil Nadu, India; 4 Department of Food Engineering, Faculty of Agriculture, Ataturk University, Erzurum 25240, Turkey; 5 Food Chemistry Laboratory, Department of Chemistry, National and Kapodistrian University of Athens Zographou, 157 84 Athens, Greece; 6 State Key Laboratory of Food Science and Technology, Jiangnan University, Wuxi 214122, China; 7 International Joint Laboratory on Food Safety, Jiangnan University, Wuxi 214122, China; 8 Human Nutrition Department, College of Health Sciences, QU Health, Qatar University, Doha 2713, Qatar

**Keywords:** phytochemicals, bioactive compounds, extraction methods, solvents

## Abstract

Recent scientific studies have established a relationship between the consumption of phytochemicals such as carotenoids, polyphenols, isoprenoids, phytosterols, saponins, dietary fibers, polysaccharides, etc., with health benefits such as prevention of diabetes, obesity, cancer, cardiovascular diseases, etc. This has led to the popularization of phytochemicals. Nowadays, foods containing phytochemicals as a constituent (functional foods) and the concentrated form of phytochemicals (nutraceuticals) are used as a preventive measure or cure for many diseases. The health benefits of these phytochemicals depend on their purity and structural stability. The yield, purity, and structural stability of extracted phytochemicals depend on the matrix in which the phytochemical is present, the method of extraction, the solvent used, the temperature, and the time of extraction.

## 1. Introduction

Phytochemicals are plant-based bioactive compounds produced by plants for their protection. They can be derived from various sources such as whole grains, fruits, vegetables, nuts, and herbs, and more than a thousand phytochemicals have been discovered to date. Some of the significant phytochemicals are carotenoids, polyphenols, isoprenoids, phytosterols, saponins, dietary fibers, and certain polysaccharides. These phytochemicals possess strong antioxidant activities and exhibit antimicrobial, antidiarrheal, anthelmintic, antiallergic, antispasmodic, and antiviral activities [1,2]. They also help to regulate gene transcription, enhance gap junction communication, improve immunity, and provide protection against lung and prostate cancers [3,4,5,6,7]. The recent focus on translational research has enhanced the dimensions of functional foods. Phytochemicals, after extraction from various sources, find profound application in the development of functional foods and nutraceuticals. Phytochemicals exhibit variations in their affinity for solvents and tolerance to heat. The selection of the solvent also affects the quality of the recovered phytochemical and its application in the development of food and nutraceutical products. The solvents can be divided into green solvents [water, ethanol, glycerol, fatty acids/oils, acetic acid, ionic liquids, carbon dioxide (CO_2_), deep eutectic solvents and natural deep eutectic solvents (NADES), etc.] and other solvents such as acetone, chloroform, butanol, methanol, ethyl acetate, methyl acetate, benzene, hexane, cyclohexane, etc. [8]. Loss in functional properties can occur with the use of non-compatible solvents and varied exposure to different temperatures. Additionally, extraction efficiency depends upon the matrix in which the phytochemical is present. Several matrix-related characteristics, such as matrix type, structure, pre-treatment, particle size, and solid–liquid ratios influence the extraction efficiencies of phytochemicals and extraction techniques [9]. To ensure quality products, phytochemicals must be extracted from the source crop in a manner that retains their natural structure and properties. Hence, it is imperative to select a suitable method of phytochemical extraction. Some of the widely used conventional methods are maceration, percolation, decoction, reflux extraction, and Soxhlet extraction, and the novel methods are pressurized liquid extraction (PLE), high hydrostatic pressure extraction (HHP), microwave-assisted extraction (MAE), ultrasound-assisted extraction (UAE), pulsed electric field extraction (PEF), vibro-cavitation extraction, extraction under vacuum-oscillating boiling conditions, extractions in mills, extraction in rotary-pulsation apparatus (RPE), liquid gas extraction (LGS), enzyme-assisted extraction (EAE), supercritical fluid extraction (SFE), and natural deep eutectic solvent extraction (NADES) [8,10]. This article discusses the suitability of phytochemical extraction methods under different extraction conditions such as substrate type, type of matrix, the solvent used, extraction temperature, etc. This will help researchers to select the right method of phytochemical extraction that will help them to develop better quality functional foods and nutraceuticals with the highest natural properties.

## 2. Methods

The articles were searched for in research engines such as Google Scholar, ResearchGate, ScienceDirect, and PubMed. Synonyms and alternative words were identified and used to obtain the current literature. The major search terms and key words used were phytochemicals, phytosterols, major bioactive compounds in plants, carotenoids, isoprenoids, saponins, anthocyanins, flavonoids, dietary fiber, polysaccharides in plants, health benefits of phytochemicals, solvents for the extraction of phytochemicals, methods of extraction, Soxhlet extraction, percolation, reflux extraction, PLE, MAE, HHP, EAE, SFE, and NADES. Only articles published in English were used. Most of the research articles included in this study were published after 2015; however, important articles published between 2000 and 2015 with significant results that could not be replaced with new studies were also added and cited.

## 3. Overview of Major Phytochemicals and Related Health Benefits

The type and concentration of phytochemicals in the source crop vary according to intrinsic and extrinsic factors such as crop type, variety, soil, and environment (region, altitude, and season) of cultivation. This section discusses major phytochemicals, their characteristics, and associated health benefits. A detailed list of major phytochemicals, their sources, and their health benefits is also provided in Table 1.

### 3.1. Carotenoids

Carotenoids are bright yellow, red, and orange-colored pigments found in plants, algae, and photosynthetic bacteria. Fruits are explicitly rich in carotenoids, whereas vegetables such as sweet potato, carrot, pumpkin, and spinach also possess carotenoids in profuse amounts [66,67,68]. They are abundantly found in carrots (*Daucus carota* L.), tomatoes (*Solanum lycopersicum* L.), parsley *(Petroselinum crispum* L.), orange (*Citrus sinensis* L.), daikon radish (*Raphanus sativus* L.), cabbage (*Brassica oleracea* L.), spinach (*Spinacia oleracea* L.), fenugreek (*Trigonella foenum-graecum* L.), round purple turnip (*Brassica rapa var. rapa* L.), and green leafy vegetables. The commonly found carotenoids are α-carotene, β-carotene, β-cryptoxanthin, lutein, zeaxanthin, lycopene, and fucoxanthin [10]. β-carotene is the most abundant carotenoid in most fruits and vegetables, followed by α-carotene; β-cryptoxanthin is a major carotenoid in tangerines, persimmons, and oranges; lutein and zeaxanthin are found mainly in green leafy vegetables; lycopene is found in tomatoes; and fucoxanthin is found in brown algae. Among these, α-carotene, β-carotene, and β-cryptoxanthin are the precursors of Vitamin A, while lutein, lycopene, and fucoxanthin are strong antioxidant agents. Lutein also plays an important role in vision. Zeaxanthin is a fat-soluble pigment and antioxidant that is concentrated in the macula region of the retina and is responsible for fine-feature vision [15]. The other health benefits of carotenoids include gene transcription regulation by lutein, α-carotene, and β-carotene [7]; enhancement of gap junction communication by β-carotene [4]; improvement of immunity by β-carotene and lutein [6]; and protection against lung and prostate cancers by α-carotene, β-carotene, lycopene, and zeaxanthin [3,5]. Fucoxanthin is reported to exhibit anticancer, antihypertensive, anti-inflammatory, radioprotective, and antiobesity effects [69].

### 3.2. Polyphenols

Polyphenols are a category of natural compounds with phenolic structures. This family has four major subclasses, such as flavonoids, stilbenes, phenolic acids, and lignans. Flavonoids are further classified as flavanones, flavones, flavonols, and anthocyanidins. Polyphenols are abundantly found in artichoke (*Cynara cardunculus var. scolymus* L.), spinach (*Spinacia oleracea* L.), broccoli (*Brassica oleracea var. italica* L.), chicory (*Cichorium intybus* L.), flax (*Linum usitatissimum* L.), onion (*Allium cepa* L.), apple (*Malus domestica* L.), plum (*Prunus subg. Prunus* L.), pear (*Pyrus* L.), grape (*Vitis vinifera* L.), and cherry (*Prunus avium* L.). Beverages such as olive oil, tea and red wine are considered good sources of polyphenols [70]. Flavanones have almost 350 aglycones and 100 glycosylate forms where a flavan nucleus is formed of two aromatic rings linked through a dihydropyrone ring [71]. Flavones represent a large group of flavonoids where the presence of a double bond between C-2 and C-3, as well as the attachment of the B ring to C-2, distinguishes these compounds [72]. Flavonols have a double bond between C-2 and C-3 and they differ from flavanones by the hydroxyl group at the third position [73]. Anthocyanidins are mostly found in nature as their sugar-conjugated derivatives anthocyanins, which are responsible for the red, blue, and purple colors found in fruit and floral tissues [74]. Health benefits of polyphenols include action against free radicals; protective effects against cardiovascular diseases, cancers, and other age-related diseases; and prevention of inflammation and allergies [23,25,31,75,76]. Flavonoids have been also found to be beneficial in angina pectoris, cervical lesions, chronic venous insufficiency, dermatopathy, diabetes, gastrointestinal diseases, lymphocytic leukemia, menopausal symptoms, rhinitis, traumatic cerebral infarction, etc. [22].

### 3.3. Isoprenoids

Isoprenoids, also known as terpenoids, are a class of natural compounds such as terpenes, sesquiterpenes, limonoids, ubiquinone, menthol, and camphor. They are organic compounds with two or more hydrocarbons arranged in a specific pattern. These are found in poplar (*Populus alba* L.), oaks (*Quercus suber* L.), eucalyptus (*Eucalyptus* L.), turpentine tree (*Syncarpia glomulifera* L.), juniper (*Juniperus communis* L.), lime (*Citrus latifolia* L.), orange (*Citrus sinensis* L.), and cannabis (*Cannabis sativa* L.) [2,31,77]. Limonene, myrcene, and pinene are some of the isoprenoids present in plants. Limonene is the most common monoterpene present in aromatic plants and fruits and imparts lemon-like flavor and aroma [31]. Myrcene is an alkene natural hydrocarbon that is an acyclic monoterpene compound. It is also known as the active sedating principle of hops and lemongrass [78]. Isoprenoids are useful in the reduction of appetite, stress, and anxiety, support digestion, have antioxidant potential, are effective in Alzheimer’s disease, promote sleep time, and help in pain relief [79]. 

### 3.4. Phytosterols

Phytosterol is the collective name of plant sterols and stanols that regulate the physiological functions associated with plants. They are abundant in olive oil and the oils of corn (*Zea mays* L.), sesame (*Sesamum indicum* L.), sunflower (*Helianthus annuus* L.), peanuts (*Arachis hypogaea* L.), macadamia (*Macadamia tetraphylla* L.) nuts, beans (*Phaseolus vulgaris* L.), and almonds (*Prunus dulcis* L.) [37]. Some of the plant sterols are campesterol, sitosterol, and stigmasterol while plant stanols are campestanol, sitostanol, and stigmastanol. Campesterol is the simplest sterol except for the 5–6 double bonds in the B-ring, having the hydroxyl group in position C-3 of the steroid skeleton and saturated bonds throughout the sterol structure. It has a methyl group present at C_24_ [80]. Sitosterol is a phytosterol that has stigmast-5-ene replaced by a beta-hydroxy group at position 3. It has an effect on benign prostatic hyperplasia (BPH), improves urinary flow rate, and has anti-inflammatory and anti-androgenic properties [81]. Stigmasterol is a steroid group identified by its hydroxyl group in C-3 of steroid structure and the presence of unsaturated bonds in the fifth and sixth positions of the B-ring. It has anti-inflammatory, antioxidant, antiosteoarthritis, anticancer, and cholesterol-lowering properties [82]. Campestanol is a 3-beta-sterol, a hydride derived from 5-alpha-campestane [38]. Sitostanol is a plant stanol produced from sitosterol that has been demonstrated to lower serum cholesterol levels by limiting cholesterol absorption [83]. Stigmastanol is a steroid group with a hydroxyl group in C-3 of steroid structure and saturated bond in the fifth and sixth positions of the B-ring. It is a 3-hydroxy steroid formed from a hydride of a 5-alpha-stigmastane [84]. Health benefits of phytosterols, in general, include support for prostate health, hair growth, reduction in LDL cholesterol, and high antioxidant activity [41,43]. 

### 3.5. Saponins

Saponins are glycosides present in plants that consist of sapogenin and sugar moieties [85,86] and are classified into steroidal saponins and triterpenoids saponins based on the nature of the aglycone. These are abundant in the legumes viz. black gram (*Vigna mungo* L.)*,* garden pea, (*Pisum sativum* L.)*,* pigeon pea (*Cajanus cajan* L.)*,* and common bean (*Phaseolus vulgaris* L.). Some of the saponins are dammaranes, tirucallanes, and oleanane. Dammarane is a tetracyclic triterpene present in sapogenins, which create triterpenoid saponins. It was first isolated and named after dammar resin, a natural resin found in tropical trees of the Dipterocarp family [87]. Oleanane is a triterpenoid found in nature. It belongs to the oleanoid family, which includes pentacyclic triterpenoids (including beta-amyrin and taxerol) with six-membered rings [87,88]. Tirucallane is a tetracyclic triterpenoid saponin mostly found in euphorbia [44]. These exhibit hypoglycemic, virucidal, antifungal, antimicrobial, and hypolipidemic activity. The impact of saponins on acute impact injuries, erectile dysfunction, venous edema in chronic deep vein incompetence, and systemic lupus erythematosus has been also reported [22]. The effect of saponins at a lower concentration of 10 µg/mL on the transverse tubular system and sarcoplasmic reticulum was also observed to be effective [48]. 

### 3.6. Polysaccharides and Dietary Fibers

Polysaccharides are a group of monomer sugar units linked by glycosidic linkage. They may be storehouses of energy such as starch and glycogen or non-digestible components such as cellulose, pectin, beta-glucan, hemicelluloses, resistant starch, lignin, etc., that are collectively known as dietary fiber. These components are not digested by human digestive enzymes [89] but are broken down by the gut microbiota in the large intestine, where they selectively support the growth of healthy microorganisms. All plant-based foods are good sources of dietary fiber and some rich sources are carob beans (*Ceratonia siliqua* L.), chicory, tamarind (*Tamarindus indica* L.), Jerusalem artichoke (*Helianthus tuberosus* L.), barley (*Hordeum vulgare* L.), corn, oats (*Avena sativa* L.), wheat (*Triticum aestivum* L.), and green beans (*Phaseolus vulgaricus* L.) [58]. The regular consumption of dietary fiber helps to prevent cancer, inflammation, hypertension, hyperlipidemia, hypercholesterolemia, obesity, and cardiovascular diseases, as well as improving insulin sensitivity and promoting healthy microbiota in the gut [61,90]. In addition, dietary fibers can improve textural and health properties, reduce cooking loss and production costs, and be used as a fat substitute in health food [91,92,93,94,95]. 

## 4. Phytochemical Extraction Methods

Extraction is defined as a process of removing or obtaining the desired compounds from the source material [96]. The solvents used for the extraction of phytochemicals can be divided into green solvents such as water, ethanol, glycerol, fatty oils, ionic liquids, acetic acid, isopropanol, supercritical CO_2_, deep eutectic solvents, natural deep eutectic solvents, etc., and other organic solvents such as acetone, chloroform, butanol, methanol, ethyl acetate, methyl acetate, benzene, hexane, cyclohexane, etc. The green solvents are termed so due to their nontoxic, biodegradable, recyclable, and renewable nature. These solvents also have a high flash point. Among the green solvents, water is the most used and universal solvent. It is a non-selective solvent and can separate all the hydrophilic substances such as saponins, phenolics, polysaccharides, etc. The extraction efficiency of water can be enhanced by superheating, as superheating decreases the dielectric constant of water and provides better penetration. Superheated water is also a better solvent for the extraction of lipophilic substances such as essential oils, as the polarity of water decreases significantly between 100–374 °C. Ethanol is selective in action and is used for the extraction of polyphenols and triterpenes. The extraction efficiencies of ethanol can be modified by using water and acid. Glycerol has high thermal stability (boiling point 290 °C). It is too viscous at low temperatures and hence has a low solubility. It can be used as an extraction solvent above 60 °C or with other co-solvents. Glycerol is not a good solvent for hydrophobic compounds such as fatty acids and oils but is a selective solvent for polyphenolic extraction. Fatty oils are a good solvent for the extraction of hydrophobic substances and can be used for the extraction of carotenoids, coumarins, tocopherols, flavonoids, etc. The major oils used for extraction are soybean, almond, olive, sunflower, etc. Acetic acid buffer can be used for the extraction of phenolics and anthocyanins. Isopropanol is a green alternative to n-hexane. It can be used for the extraction of oils, alkaloids, gums, and natural resins. Supercritical CO_2_ is used for the extraction of lignans, anthocyanins, and essential oils. Ionic liquids are non-volatile, non-inflammable salts with low melting points (below 100 °C). The melted salts form a liquid that is composed of ions that have high thermal stability, high conductivity, high heat capacity, low flammability, and low or negligible vapor pressure. These can be used for the extraction of a wide range of organic and inorganic compounds such as flavonoids, alkaloids, saponins, lignans, etc. Deep eutectic solvents are a mixture of two or more pure compounds having a eutectic point temperature below an ideal liquid mixture. Deep eutectic solvents are used for the extraction of alkaloids, flavonoids, saponins, and phenolic compounds, and the most commonly used deep eutectic solvents for the extraction of phytochemicals are type III (choline chloride and urea in ratio 1:2) and type IV (choline chloride and zinc chloride in ratio 1:2). NADES solvents are made up of natural metabolites such as organic acids, amino acids, sugars, polyols, and choline derivatives. These solvents have a low volatility and melting point and a broad polarity range. These solvents can be used for the extraction of compounds that are poorly soluble in water. Among the organic solvents, acetone has low toxic potential and is a suitable solvent for the extraction of alkaloids, oils, etc. Ethyl acetate is non-toxic and is used for the extraction of flavonoids, total phenolics, etc. Methanol and chloroform have inherent toxicity. Methanol is used for the extraction of flavonoids, saponins, tannins, etc. Chloroform is used with other solvents such as ethanol and fatty acids for the extraction of alkaloids and anthocyanins. Butanol can be used for the extraction of saponins, total phenolics, and flavonoids; however, it is mainly used for the purification of fractions of individual compounds. n-hexane is a solvent with low acute toxicity. It can be used for the extraction of flavonoids, carbohydrates, anthra-glycosides, and saponins. Methyl acetate is a volatile solvent that is produced by acetic acid esterification with methanol or as a byproduct during methanol carboxylation. It is used mainly for the extraction of phytosterols and tocopherols. Benzene is a potentially dangerous chemical. It can be used for the extraction of flavonoids, phytosterols, alkaloids, and volatile oils. Cyclohexanes are used for the extraction of fats, waxes, and oils [10,97,98,99]. The detailed considerations for the suitable solvent for the extraction of phytochemicals have been discussed by Kim and Wijesekra [69].

The extraction efficiency can be further enhanced by optimizing extraction conditions such as the choice of solvent, temperature, and time. The extraction process starts with cell lysis, followed by the collection of extract, isolation, purification using chromatographic techniques to separate bioactive compounds from the mixture, and identification of phytochemicals using spectrophotometry [100,101,102,103]. Various extraction methods and their mechanism of action are discussed in detail in this section and are also provided in Table 2. 

### 4.1. Maceration

**Figure 1:**
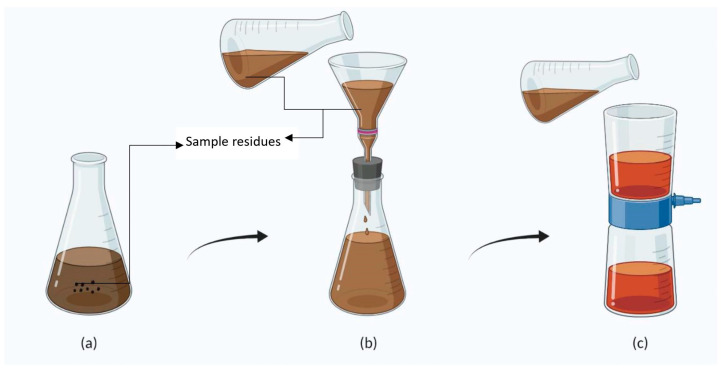
A diagrammatic representation of maceration extraction. (**a**) Sample and solvent mixed and kept for 7 days. (**b**) Straining of liquid and pressing the remaining marc. (**c**) Filtering out more clear liquid.

The term “maceration” means softening. It is used to make tinctures, extracts, and concentrated infusions. In this method, the sample is soaked in a solvent/menstruum (aqueous and non-aqueous) such as water, ethanol, methanol, petroleum ether, etc. (Figure 1), for 3–7 days with occasional shaking to extract the phytochemicals [135]. After maceration, the liquid is strained out, and the solid residue (Marc) is pressed to extract as much liquid as possible. This is followed by filtration or decantation to clarify the liquids [136,137]. Infusions are prepared for soft tissues such as petals, leaves, flowers, etc. The standard conditions for the preparation of infusions are 15 min of maceration in a water bath and 45 min of cooling to room temperature [8]. Various phytochemicals such as quinones, flavonoids, polyphenols, tannins, terpenoids, alkaloids, polypeptides, steroids, saponins, coumarins, and glycosides can be extracted using this method. This extraction method is a safe and effective process for crude drug extraction and is also recognized by the Indian Pharmacopoeia for the extraction of crude drugs [136]. The extraction efficiency depends on the type of solvent, extraction time, extraction temperature, and food matrix. Maximum extraction with a minimum amount of solvent can be achieved with this method [138]. Glycosides, alkaloids, terpenoids, saponins, and carbohydrates can be best extracted using water; ethanol is a good solvent for glycosides, carbohydrates, alkaloids, and quinines; methanol gives better extraction for flavonoids, tannins, glycosides, phenolic compounds, and amino acids; acetone is best suited for alkaloids, glycosides, sterols, and saponins; ethyl acetate is suitable for terpenoids, glycosides, quinones, and carbohydrates; and petroleum ether gives higher extraction efficiencies for amino acids [139]. Low polar solvents such as acetone have a higher recovery rate (100%) compared to intermediate polar solvents (ethyl acetate–87–94%) and highly polar solvents (ethanol–82–88%). Extraction solvents and their polarity are discussed in Table 3. Extraction without using high temperature helps in extracting thermolabile compounds. Generally, a maceration time of 2–3 days at a temperature of 20–25 °C is best preferred [140]. 

### 4.2. Percolation

**Figure 2:**
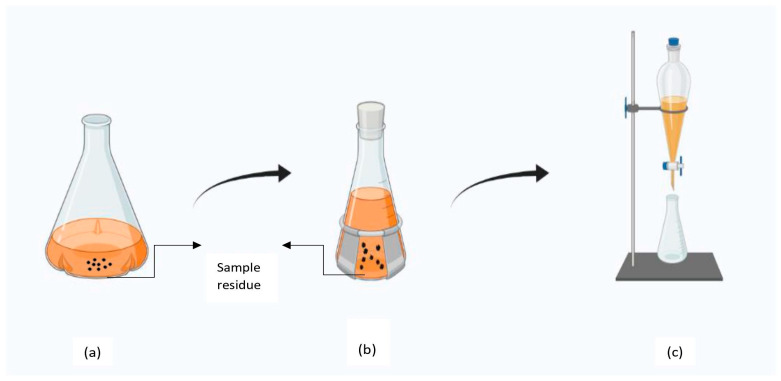
A diagrammatic representation of percolation extraction. (**a**) Sample immersed in solvent. (**b**) Sample placed in a sealed decanter for 4 h. (**c**) Filtering out using a percolator.

Percolation is widely used in the extraction of essential oils from seeds, bark, and leaves of plants. To perform the extraction, the sample is immersed in a surplus volume of menstruum, and then it is transferred into a sealed decanter for 4 h, as shown in Figure 2. After that, it is carefully inserted into the percolator ensuring an even aisle and complete fluid contact, and the percolator is filled with liquid and capped. The bottom outlet is opened until a standard drop rate or percolation rate (the speed at which the sample moves through different layers in a percolator) is achieved. Following this, more menstruum is added to soak all of the material in the percolator for 24 h. The wet mass so obtained is pressed to extract the maximum residual fluid and supplied with enough solvent to obtain the desired proportion [137]. The extraction rate in percolation is influenced by the type of solvent and drop rate as it determines the solvent’s flow rate. The temperature of extraction should be selected as per the desired phytochemical [141]. The recovery of the extract also varies with the concentration of solvent and the part of the plant used for extraction. Solvents such as ethyl ether and hexane have higher efficiency in extracting oil from mustard and sunflower seeds [107]. Aqueous extraction (5–7%) is more efficient for extracting phytochemicals from leaves of *Syzygium cumini* L. [142]. The major limitation of this method is its high solvent consumption and long extraction times as it requires a high substrate-to-solvent ratio and four to five extraction repetitions (1–2 days) are required [143]. An advancement in the percolation process is repercolation or repeated percolation. In this, many percolators (3–12) are connected through a pipeline in series and the solvent and the sample operate in a counterflow manner as the fresh solvent comes in contact with exhausted plant material [8].

### 4.3. Decoction

**Figure 3:**
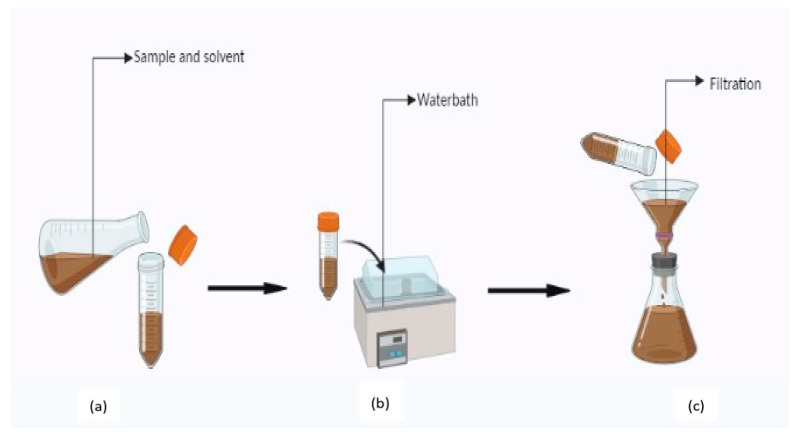
A diagrammatic representation of decoction extraction. (**a**) Sample placed in a capped glass tube, then a (**b**) water bath for 65–70 °C for 30 min to 2 h. (**c**) Filtration of the extract.

Decoction is a method used to extract heat-stable, oil-soluble compounds such as saponins, tannins, and flavonoids from hard plant materials such as roots, bark, and stem [144]. In this method, plant material is cooked for 30 min to 2 h in a sufficient quantity of water and then strained through layers (probably eight) of muslin cloth (Figure 3). The process can be coupled with centrifugation for a better extraction, from which the solvent is evaporated after collecting the supernatant [142]. Normally, extraction is carried out at a temperature of 65–70 °C for almost 2 h and the temperature is maintained throughout the process [109]. The decoction is strongly affected by pH, temperature, and the number of herbs. When compared with maceration, the decoction method may improve the solubility of several phytochemicals *viz.* alkaloids, flavonoids, soluble polysaccharides, and tannins [144]. The high temperature used in the decoction process inhibits the action of glucuronidase, preventing glycosides from being converted to their aglycones. This leads to the detection of higher levels of flavones (Baicalin and Wogonin) in the extracted decoctions [135]. The major limitations of decoction are the high level of impurities present in the crude extract. The extraction of heat-sensitive or volatile components cannot be performed with decoction [144].

### 4.4. Reflux Extraction

**Figure 4:**
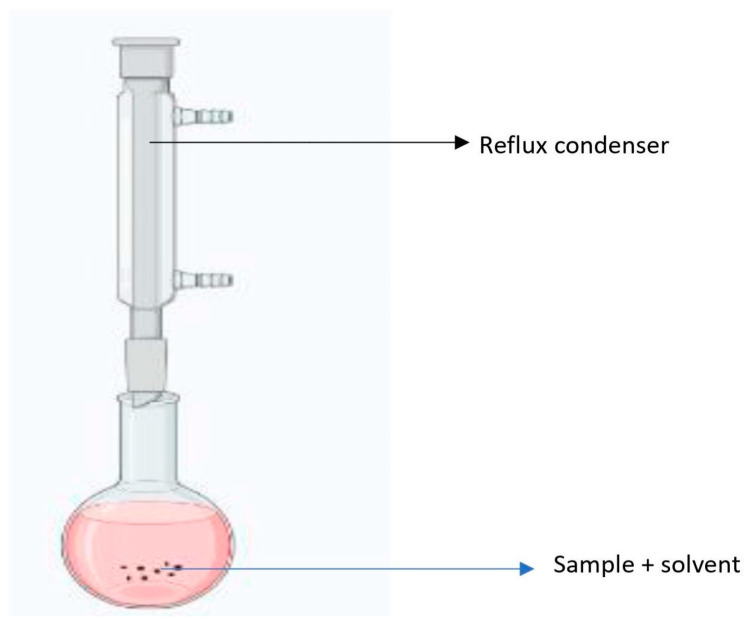
A diagrammatic representation of reflux extraction.

Reflux extraction is the solid–liquid extraction method, as shown in Figure 4, where solvent evaporation and condensation are carried out for 1–3 h at a constant temperature (60–80 °C) [111]. The reflux method is widely used for the extraction, quantification, and evaluation of various phytochemicals and essential oils from plant sources. The average extraction temperature and time using mixed solvents (water and ethanol) have been reported to be 60–80 °C and 80–120 min, respectively [112]. The extraction efficiency of this method varies from 70 to 90% [145]. The boiling point of the solvent and extraction time are the two most important regulating factors in this approach. Depending on the criteria being followed, extraction should continue until asymptotic levels or exhaustive circumstances are attained. This method provides fine control over the temperature and hence can prevent the loss of organic compounds [114,144]. The major limitation of this method is its unsuitability for the extraction of thermolabile components [144]. 

### 4.5. Soxhlet Extraction

**Figure 5:**
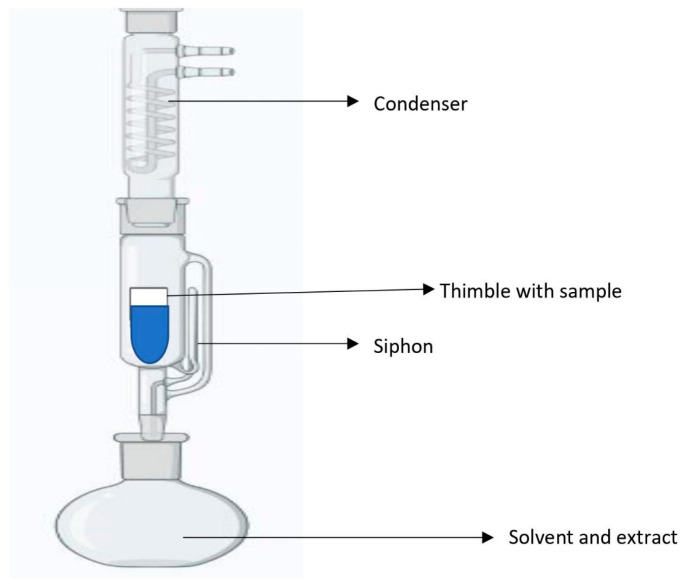
A diagrammatic representation of Soxhlet extraction.

In Soxhlet extraction, a small amount of dry material is inserted in a thimble (Figure 5), which is then placed in a distillation flask holding solvents such as toluene, hexane, petroleum ether, etc. [146]. It is a high-efficiency automatic continuous extraction technology that consumes less time and solvent than maceration or percolation. High temperatures and long extraction periods increase the chances of thermal deterioration. The Soxhlet extraction process includes the benefits of both reflux and percolation, using refluxing and siphoning principles to constantly extract with fresh solvent. The time taken for the extraction is nearly 24 h at 65–100 °C [147]. Several factors such as the selection of plant material, drying rate, size reduction of sample, temperature, and selection of solvent influence this extraction. Drying is critical for extraction, as fresh plant materials contain active enzymes and excess water can degrade the quality of phytochemicals. Soxhlet extraction also requires grinding and size reduction, as the smaller the particle size, the more the surface area and the greater the extraction rate. Inert and easy-to-remove solvent should be used for extraction. The order of acetone, petroleum ether, ethyl acetate, chloroform, methanol, ethanol, and water, for example, is used to pick solvents with increasing polarity (Table 3). Petroleum ether is often used for the extraction of steroids and essential oils, removal of chlorophyll from leaf powder, and defatting plant materials. Methanol is employed for the extraction of terpenoids, steroids, flavonoids, and glycosides. Tannins, saponins, and carbohydrates are extracted using chloroform, acetone, and ethyl acetate [148,149]. Samples with high water content cannot be extracted using this method as it might cause the degradation of flavonoids. The prolonged extraction process and the high volume of solvents also make this process expensive. The disposal of the solvent after use also raises environmental concerns. The high extraction temperatures may also result in a thermal breakdown of the target molecule [144,150]. 

### 4.6. Pressurized Liquid Extraction

**Figure 6:**
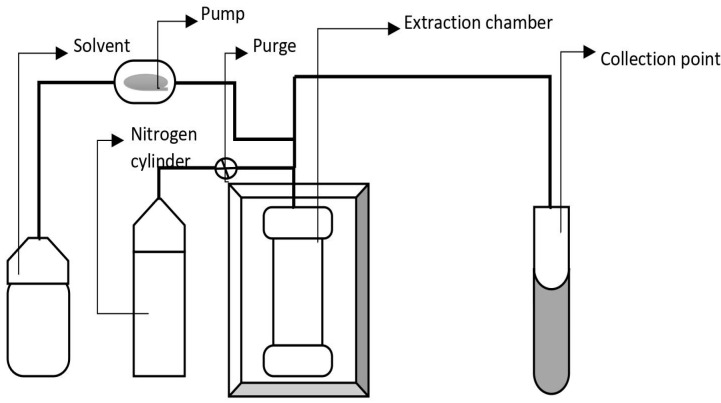
A diagrammatic representation of pressurized liquid extraction.

Pressurized liquid extraction (PLE) uses elevated temperature and pressure to extract analytes from a solid matrix quickly and efficiently using liquid solvents. It exhibits high solubility and mass transfer properties [151]. Enhanced solvent extraction (ESE), accelerated fluid extraction (ASE), and high-pressure solvent extraction (HSPE) are all terms used to describe PLE. PLE uses high pressure to retain solvents in a liquid form beyond their boiling point, allowing for easier extraction, as shown in Figure 6. The high temperature and pressure used in this method significantly reduce solvent requirement and extraction time. The extraction time in PLE varies from 5–20 min and the extraction efficiency varies from 95–100%. The temperature and pressure at which extraction is carried out vary from 50–200 °C and 50–300 psi, respectively [115,116]. In comparison to Soxhlet, PLE uses considerably low quantities of solvent and extraction time. PLE is a potential alternative for supercritical fluid extraction (since CO_2_ is not a good solvent to extract polar compounds) to extract bioactive compounds from *Cacospongia mycofijiensis* (oceanic sponge) [152]. This extraction method is thought of as an organic version of supercritical fluid extraction, with organic solvents replacing CO_2_. The solvent requirement in this method is very low and, hence, it is also known as a green extraction method. Extraction efficiency depends on the extraction pressure, temperature, and surface tension of the solvent. The application of high pressure, high temperatures, and low solvent surface tension allow better penetration and better extraction rates. Non-ionic surfactant solutions have recently emerged as a viable alternative to ionic surfactant solutions in PLE [151]. PLE has been efficiently used for extracting paraben, bronidox, and triclosan, which are allergens and also used as cosmetic preservatives. Gas chromatography coupled with PLE helps in the extraction of pyrethroid and organophosphorus residues in herbal plants such as tea. Antibiotics and other bioactive compounds linked with suspended solid matter can be extracted using PLE. A completely automated PLE approach can be used for the multi-residue analysis of sulfonamide antibiotics in soils [151,153]. A high level of extract dilutions (when using multi-cycle extraction), low analyte selectivity, presence of interferents, and advanced instrumentation requirements are the major limitations in PLE [154]. The higher flow rate of solvent leads to excess dilution of extract, which may require an extra step of concentration [155].

### 4.7. Liquid Gas Extraction

Liquid gas extraction (LGE) is a batch or semi-continuous process of extraction that is performed using solvents such as liquefied petroleum gas (a mixture of n-propane and n-butane) or dimethyl ether at room temperature or low pressure (200–1000 kPa). The extraction is performed in an extractor vessel that contains the solid sample. The solvent used for the extraction can be fed from either top or bottom and it is circulated between the tanks until the extraction is complete. The flow rate of solvent is controlled by a needle valve, the temperature is controlled by heat exchangers, and the extract is collected in an extract storage tank connected in series. The extraction can be carried out in isobaric and non-isobaric modes. Under isobaric conditions, the solvent keeps recirculating without a pump or compressor due to constant pressure, whereas, under non-isobaric conditions, the liquefied gas flows through the raw material using a circulating pump. On completion of the extraction, the extract is fed to a separator, where the expanded volume reduces the pressure and, because of its low boiling point, the gas is flashed off. Extraction using this method can be performed at a pressure just above 5 bar and, hence, this method has an advantage over the use of CO_2_ in liquid gas extraction that requires a pressure of 150–500 bar. This technology can be used for the extraction of lipids, carotenoids, terpenoids, etc. The use of liquefied petroleum gas for the extraction of phytochemicals has been reported to cause less decomposition or modification of compounds [10,131]. The major limitation in the use of this technology is its expensive nature, as the storage and transportation of the solvent require high pressure and low temperature to maintain it in the liquid state, thus requiring special trucks for transportation and special structures for storage. 

### 4.8. Microwave-Assisted Extraction

**Figure 7:**
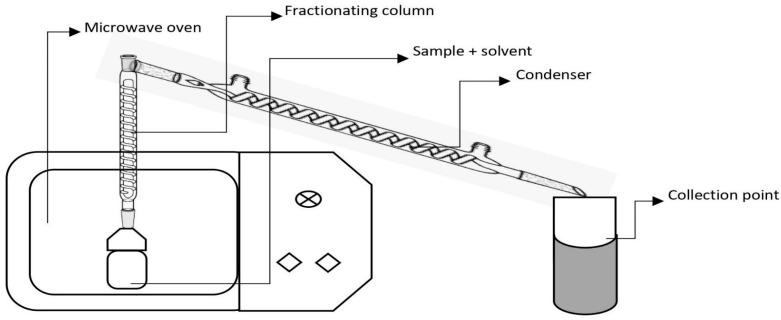
A diagrammatic representation of microwave-assisted extraction.

Microwaves are high-frequency electromagnetic waves placed between the radiofrequency and far infrared (IR) regions of the electromagnetic spectrum, corresponding to a frequency range from 0.3 to 300 GHz and wavelengths from 1 mm to 1 cm [156]. In this extraction method, plant metabolites are extracted by utilizing strong microwaves along with solvents, as shown in Figure 7. Microwave extraction in food analysis allows food to be dried, extracted, and condensed using just one piece of equipment and a small amount of sample. MAE has also been used in food ingredient analysis, including flavor composition, pectin from waste mango peel, amino acids in vegetables, and fatty acids from vegetable oils and animal fats [157]. In MAE, inside-out heating occurs, which affects molecules by ionic conduction and dipole rotation. The two frequencies at which microwave devices operate are 915 and 2450 MHz. At 915 MHz, large ablation zones are produced with a high penetration depth of EM energy [158]. At 2450 MHz, the dipoles align and randomize 4.9 × 10^9^ times per second. This forced molecular movement results in molecular friction and heating of the solution [159]. MAE rapidly heats the sample solvent mixture, resulting in wide applicability for the rapid extraction of analytes, including thermally unstable substances. In closed-vessel MAE, the temperature attained during extraction (for solvents such as acetone, acetone–hexane, dichloromethane–acetone) is 2–3 times the boiling point of the solvents, resulting in improved extraction efficiencies from the sample matrix. MAE’s effectiveness is determined by several factors, including solvent characteristics, sample material, the components being extracted, and their dielectric constants. Kapoore et al. [160] observed an extraction time between 30 s to 20 min at a temperature of 120–140 °C. Solvents with strong microwave-absorbing capacity, such as methanol, ethanol, and water, can easily heat up, lowering the duration of microwave power application time, and should thus be employed in the processes. The limitation of MAE is that it has the potential to damage polyphenols with many hydroxyl-type substituents and heat-sensitive polyphenols such as anthocyanins. So, it is sparingly employed when considering polymeric polyphenols such as anthocyanins and tannins [161].

### 4.9. Ultrasound-Assisted Extraction

**Figure 8:**
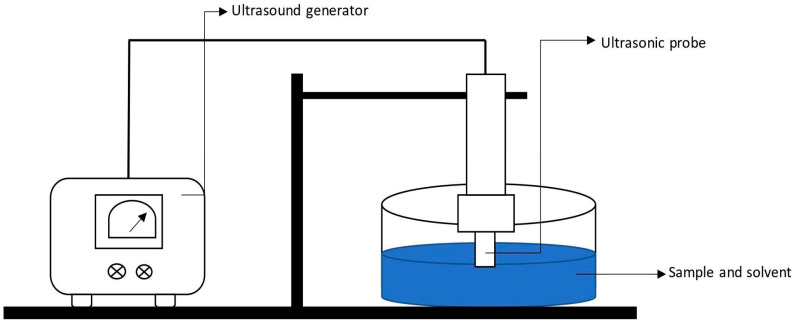
A diagrammatic representation of ultrasound-assisted extraction.

Ultrasonic extraction or ultrasound-assisted extraction (UAE) is based on the principle of acoustic or ultrasonic cavitation. Acoustic coupling occurs from high-power ultrasonic waves that are coupled using a probe-type ultrasonic processor into the slurry, where these travel through the liquid, creating alternating high-pressure or low-pressure cycles (Figure 8). In UAE, mechanical energy generated by ultrasound waves results in cavitation [161]. This achieves complete extraction and yields superior extracts in a very short extraction time in a cost-effective manner. This results in high-quality extracts that are used for food, pharmaceuticals, and supplements [119]. The application of ultrasound in the food industry can be divided into low-intensity high-frequency ultrasound (f > 100 kHz) and high-intensity low-frequency ultrasound (20 kHz ≤ f ≤ 100 kHz). Low-intensity ultrasound does not alter the physical or chemical properties of the material, but high-intensity shock waves generate intense pressure and temperature gradients caused by bubble cavitation, producing a disruption effect within the matrix. Acoustic/ultrasonic cavitation leads to extreme temperatures and heating/cooling rates, pressure differentials, and high shear forces in the medium. When cavitation bubbles implode on the surface of solids, micro-jets and inter-particular collision generate effects such as surface peeling, erosion, particle breakdown, cell disruption, and sonoporation, i.e., the perforation of cell walls and cell membranes. The implosion of cavitation bubbles in liquid media creates micro-mixing and macro-turbulences. UAE has an extraction efficiency of 85 to 97% depending on the type of sample being extracted at a 20–80 °C temperature range within 10–60 min [120]. The nature of the solvent and its characteristics, extraction time, temperature, power, and the presence of gases are all important factors in UAE. An increase in temperature initially improves UAE yield, but as the temperature rises above 45 °C, the yield declines. In UAE, several solvents such as acidified water, alcohols, acetone, and water can be utilized to extract various phytochemicals. Acidified water is preferred for pectin extraction [162]. To be safe and to completely avoid phenolic thermo-degradation, the recommended temperature is less than or equal to 40 °C [163]. The application of UAE for recovering phytochemicals attached to complex matrixes such as algae remains a limitation due to the lack of proper power regulation, resulting in inefficient energy transmission within the extracted vessel [9,162].

### 4.10. Pulsed Electric Field Extraction

**Figure 9:**
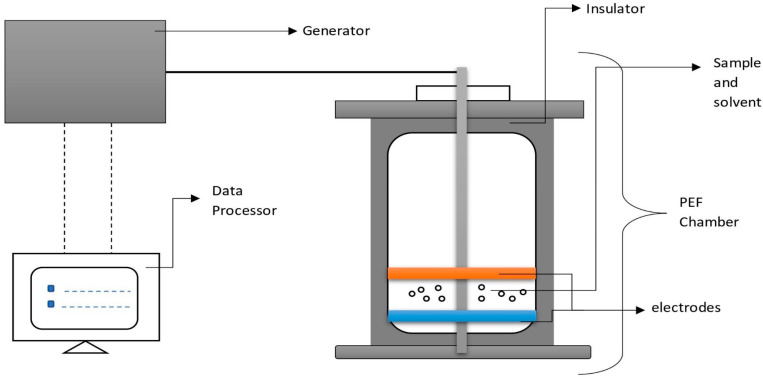
A diagrammatic representation of pulsed electric field extraction.

Pulsed electric field extraction (PEF) is a non-thermal technique of extraction in which a direct high electric current is applied using either a batch method (100–300 V/cm) or a continuous method (20–80 kV/cm) for a short time interval (nanoseconds to microseconds) to a product placed between two electrodes [121,123]. This makes the sample’s cell membrane more conductive, porous, and permeable and allows substances trapped inside the cell membrane to flow through into the surrounding fluid (Figure 9). Extraction is carried out at 20 to 50 °C with the time ranging from 5 min to 48 h. It has an estimated extract yield of about 76 to 85% [121,122]. Pulse parameters, treatment medium, and the physicochemical attributes of cells are major factors influencing the degree of electroporation in pulsed electric field extraction. The degree of electroporation may be affected by pulse characteristics such as electric field strength, pulse duration, pulse polarity, and pulse frequency. Tissue parameters such as size, thickness, structure, ionic mobility, and water content also influence the rate of electroporation [164]. PEF has been used as a non-thermal intensification approach to boost oil yield extraction from sunflower seeds. After treating sunflower seeds for the 30 s under an electric field of 7.0 kV/cm with a frequency of 15 Hz, solvent content of 40 wt percent, and a pulse width of 30 s, oil extraction increased by 9.1% [165]. PEF can be employed for the extraction of water-soluble proteins [166]. Chemical and electrochemical reactions such as electrode fouling and corrosion are considered serious drawbacks when high-intensity treatment is carried out [167]. 

### 4.11. Enzyme Assisted Extraction

**Figure 10:**
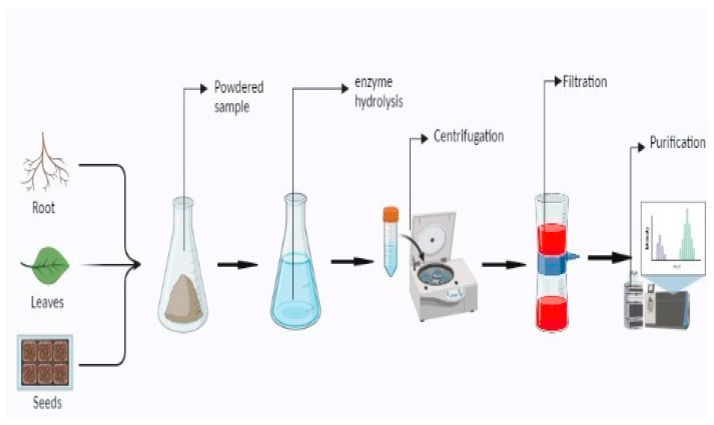
A diagrammatic representation of enzyme-assisted extraction.

Enzymes are efficient catalysts for extracting, modifying, or synthesizing complex bioactive substances from nature. The intrinsic capacity of enzymes to catalyze reactions with precise specificity and regioselectivity underpins enzyme-based extraction (Figure 10). It could be a viable alternative to traditional solvent-based extraction methods and offer a green strategy that helps to alleviate environmental problems. Cellulases, hemicellulases, and pectinases are the most-utilized enzymes for extracting bioactive compounds. Enzymes hydrolyze the cell wall and disrupt the structural integrity of plant material [168]. Most EAE is carried out in a temperature range of 33 to 67 °C and the extraction time varies from 20 to 220 min. The extraction efficiency may vary from 38 to 52% of the extract [124,125]. Enzymes have a significant impact on hydrolytic characteristics due to their composition, enzymatic activity, constituent type, and concentrations. Enzymatic activity is directly proportional to temperature. With the temperature rises, the viscosity of extraction mediums decreases and makes bioactive compounds more soluble. Enzymes normally require an acidic pH of 4.5, as it affects catalytic activity by changing protein structures and increasing substrate binding capacity [169,170]. An enzyme-assisted extraction approach, rather than acid precipitation, was found to be more effective in recovering catechins (100% yields) from diverse milk tea beverages with a yield of 74% [171]. Pectic enzymes are used extensively in food processing for the extraction of pectin, essential oils, flavors, and pigments from plant parts. Cellulase, α-amylase, and pectinase are the most commonly employed enzymes for oil extraction. When compared to hexane-extracted oils, the quality of enzyme-treated oils is relatively good [168]. The high price of enzymes is a technological limitation for the extraction of a large number of samples. The complete hydrolysis of plant cell walls is not attained with the help of enzyme combinations currently available, which limits the extraction yield of phytochemicals. The extraction rates of enzymes also vary with environmental conditions such as temperature, nutrient availability, and dissolved oxygen percentage [168]. 

### 4.12. Supercritical Fluid Extraction

**Figure 11:**
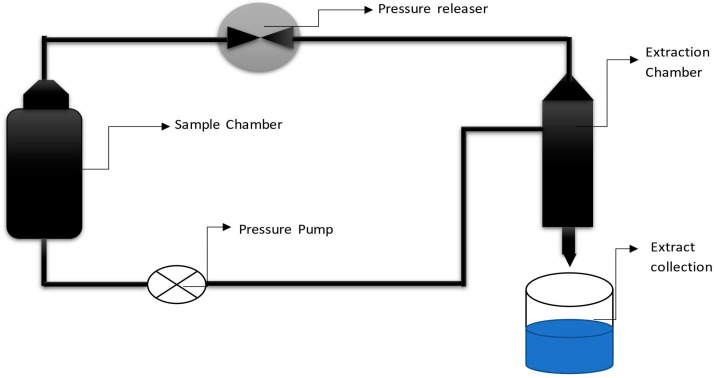
A diagrammatic representation of supercritical fluid extraction.

Supercritical fluid extraction (SFE) is commonly carried out using two steps, i.e., extraction of soluble substances from the matrix using supercritical fluid and separation or fractionation of the extracted phytochemicals from the supercritical solvent after the expansion of gas, as shown in Figure 11. SFE is based on the use of solvents above the critical temperature (40–80 °C) and pressure (10–35 MPa) [126]. SFE can be a fast, clean and efficient method for extracting natural products from several matrices [172]. The basic instruments used for SFE must be capable of withstanding high pressures (typically 50 MPa). The equipment needed is different depending on the solid or liquid samples. In countercurrent supercritical fluid extraction (CC-SFE) sample is fed from the top of the column and the solvent is pressurized from the bottom. The components are distributed between the solvent and the liquid sample, which flows in a counter-current manner through the separation column. Considering the separation factor between extraction components, the desired contact time between the sample and the solvent can be reached by adjusting the sample height in the extraction column. Among the green solvents used in SFE, carbon dioxide (31 °C and 7.38 MPa) is the most commonly employed solvent. CO_2_ is generally recognized as safe (GRAS), inexpensive, and environment friendly. Supercritical CO_2_ (SC-CO_2_) is also considered attractive because of its high diffusivity and solvent strength. CO_2_ is gaseous at room temperature and pressure, which provides solvent-free extracts [173]. Extraction pressure, temperature, time, solvent-to-material flow ratio, and solvent flow rate are important factors that influence the extraction rate. The density of the supercritical fluid will decrease as the temperature rises and this not only reduces its extraction capability, but can also cause heat-sensitive phytochemicals to degrade. With an increase in pressure at a constant temperature, the density of supercritical fluid increases, thereby increasing its extraction capacity. The higher the mass transfer rate of supercritical fluid, the higher will be the sample-to-solvent ratio and flow rate, but the faster the flow rate, the lower the resident time of solvent used [174,175]. SFE has been used in different fields such as the chemical, fuel, food, and pharmaceutical industries. Its application includes the decaffeination of coffee and the extraction of fatty acids, essential oils, and flavors from natural sources [176]. SFE is also preferred for the extraction of valuable bioactive compounds (biomolecules, flavors, and colorants) due to the absence of toxic residues in the final product [127]. The major limitations of this method are the requirement of high pressure and the low extraction yields compared to the liquid solvents. The most commonly used extraction solvents such as SC-CO_2_ are weak solvents that cause a reduction in the molecular weight range and polarity of the sample [177,178].

### 4.13. High Hydrostatic Pressure Processing

**Figure 12:**
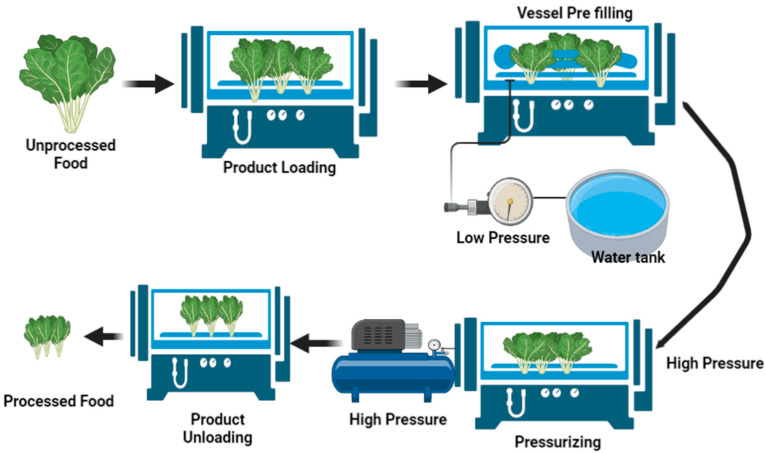
A diagrammatic representation of high hydrostatic pressure processing.

High hydrostatic pressure processing (HPP) is a non-thermal pasteurization technology used for liquid foods that inactivates harmful pathogens and vegetative spoilage microorganisms by using a pressure of about 400–600 MPa [179]. The pressure range in different applications may vary between 100–800 MPa [180] or even more, up to 1000 MPa [181]. The benefit of HPP over other processing methods is its operation at chilling or mild temperatures (usually below 45 °C) that causes minimal damage to quality attributes such as taste, appearance, and nutritional value. This technology is also used in the extraction of phytochemicals and bioactive compounds from natural biomaterials. HPP extraction is performed in a high hydrostatic vessel and the food sample to be extracted is mixed with the appropriate solvent (water is the major solvent used in industrial applications; however, ethanol, glycerol, silicon oil, or a mixture of solvents can also be used in laboratory applications), hermetically sealed in a sterile high-density polyethylene bag, and pressurized by generating pressure by pumping liquid medium into the pressure chamber. The pump is stopped upon achieving the desired pressure and the sample is maintained at this pressure for the desired holding time for the solubilization and extraction of bioactive compounds. On achieving the extraction, the system is depressurized. The high pressure disrupts the cell wall and releases the bioactive-compound-containing cytoplasm, and the bioactive compounds are removed from the solvent. This extraction method works via pressure application and, hence, the extraction efficiency is greatly influenced by pressure. The increase in pressure increases the solubility of soluble constituents [130]. Figure 12 represents the function of high hydrostatic pressure processing.

### 4.14. Natural Deep Eutectic Solvent Extraction

**Figure 13:**
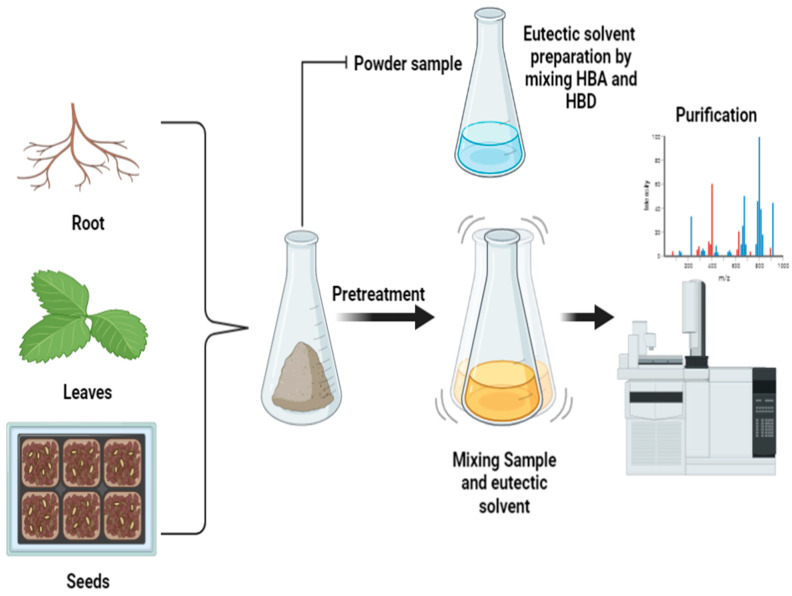
A diagrammatic representation of natural deep eutectic solvent extraction.

Natural deep eutectic solvent extraction (NADES) uses aqueous solvents such as reline, ethaline, and glycerine that are formed by mixing certain naturally available solid compounds such as sugar, amino acids, choline derivatives, alcohols, and organic acids at a specific molar concentration, which differ depending on the compound being mixed [182]. These compounds are in a solid state and become liquid when mixed in a specific combination at an appropriate molar ratio. Eutectic solvents are made with hydrogen bonding where a hydrogen bonding acceptor (HBA) is mixed with a hydrogen bonding donor (HBD), as shown in Figure 13. Three methods are mainly used: 1. Thermal mixing, where the components are mixed at about 80 °C for 1–2 h on a hot plate with magnetic stirring until a colorless clear liquid is formed; 2. vacuum evaporation, where the components are dissolved in water with a rotary evaporator before being evaporated at 50 °C; and 3. freeze-drying, where components are dissolved in water and then freeze-dried. Costa et al. [183] also prepared NADES using choline chloride–oxalic acid in a microwave system. NADES utilizes chemically tailorable solvents, as their physical–chemical properties are controllable with changes in their compositions. Other factors affecting the properties of NADESs include water content and temperature. Dilution with water reduces the density and viscosity of the NADES while increasing its polarity. Water has a bell-shaped relationship with the solubility of various solutes in NADES and the conductivity of the NADES. Decomposition temperatures for NADESs are higher than 135 °C and glass transition temperatures are lower than 50°C, with no melting points. This means that NADESs can be employed with solvents at temperatures ranging from 0 to 100 °C [184]. The stability of the mixture of solvents strongly affects the extraction rate [185,186], and an extraction efficiency of 62% has been obtained at a temperature of 25 to 105 °C and extraction times of 30 to 60 min [132,133]. For dietary fibers and polysaccharides (especially lignin), the most effective and common HBA is chloroform [187]. Mannose–dimethyl urea–water at a ratio of 2:5:5 shows higher solubility for curcumin [188]. Major limitations of NADES are the use of a mixture of different components for the synthesis of eutectic solvents and their high viscosity. Eutectic solvents are less stable and have a chance of turning back to solid when compared to other solvents. Thus, these can only be prepared at the time of extraction and cannot be held for a longer period [185]. 

### 4.15. Other Extraction Methods

The other methods are advancements or small modifications in the existing methods. The accelerated solvent extraction method is an alternative to maceration, percolation, and Soxhlet extraction. In this method, extraction is carried out at high temperatures (50–200 °C) and pressures (100–140 atm) with liquid solvents. This increases the diffusivity of the solvents. The other method is extraction in a rotary pulsation apparatus. In this system, the extraction takes place due to the combination of hydrodynamic phenomena and mechanical oscillations. The other methods of extraction are vibro-cavitation and vacuum-oscillation boiling. A vibratory ball mill also known as a tissuelyser or mixer mill can also be used for the extraction of plant materials. This mill is equipped with high-speed large amplitude arms that grind, mix, or break cells. The grinding is due to friction within the balls and the process completes within a few seconds [8]. 

## 5. Suitability of the Methods for the Extraction of Various Bioactive Compounds 

### 5.1. Carotenoids

Various methods have been adopted by scientists for the extraction of carotenoids. This section discusses the literature on the conditions and suitability of these methods. Yaqoob et al. [189] studied the extraction of carotenoids from dried ripe kinnow (*Citrus reticulata*) fruit peel using reflux extraction, UAE, and SFE with different concentrations of solvents (50%, 80%, and 100% *v*/*v*). To perform reflux extraction, 1 g dried peel was extracted with 50 mL each of ethanol, methanol, and acetone for 4 h at 30 °C. For UAE, a probe sonicator was used for 100 mL each of ethanol, methanol, and acetone, and extraction was carried out for 10 min. SFE was performed at 400 bar and 333 K with CO_2_ (flow rate of 3 mL/min) along with co-solvents (23% *v*/*v* acetone, ethanol, and methanol). The highest recovery of carotenoids (5.17 mg/100 g sample) was observed in SFE while the lowest (0.98 mg/100 g) was found in reflux extraction. Among the solvents, acetone had the highest recovery of carotenoids and β-carotene. Mihalcea et al. [190] studied the SFE of oleoresins and their carotenoids from dried seabuckthorn pomace using CO_2_. For the extraction, 400 g of sample was loaded into the extraction equipment and pressurized with 99.99% pure CO_2_ using a high-pressure pump at two different temperatures and pressure conditions, i.e., 35 °C, and 45 MPa for 105 min under the first condition and 37.5 °C and 36.5 Mpa for 105 min for the second condition. The extraction yields obtained under both conditions were 67.6 g/kg d.w. and 63.6 g/kg d.w., respectively. The total carotenoid content in the first extract was 396.12 mg/g d.w. and in the second extract it was 206.73 mg/g d.w. Ordóñez-Santos et al. [191] studied UAE of ground mandarin (*Citrus reticulata*) epicarp. The sample was mixed with 4 mL of sunflower oil at a sample-to-solvent ratio of 0.0004 g/mL and the extraction was carried out in an Ultrasonic Cleaner (HB-S49 DHT, China) at 240 W, 42 kHz, and 60 °C for 60 min. The total carotenoid content obtained was 140.7 mg/100 g d.w. sample. Purnomo et al. [181] studied the solvent extraction of carotenoid pigments from red fruit juice (*Pandanus conoideus Lam*) using maceration. The solvents used were 50 mL each of 99.5% ethanol, 99% acetone, and distilled water. Each solvent was separately mixed with 5 g of sample and kept at room temperature for 24 h in the dark. After 24 h, the sample was filtered, and optical density was measured spectrophotometrically at different wavelengths for each solvent. The distilled water extract had an absorption peak of 266 nm, which was less than the visible range. Ethanolic extract gave an absorption peak at 481 nm. Acetone extract gave an absorption peak at 476 nm. When characterized using fluorescence spectroscopy, distilled water extract gave an excitation peak at 290 and 330 nm, and emission peaks were observed at 360 and 430 nm. Ethanolic extract gave an excitation peak at 266 and 294 nm and an emission peak at 343 and 344 nm. Acetone extract gave an excitation peak at 334 and 350 nm and emission peaks at 394 and 561 nm. Since the fluorescence spectra of acetone extract exhibited emission and excitation peaks in the visible range, acetone was found to be best suited for the extraction of carotenoids. Li et al. [192] studied the extraction efficiency of lycopene and β-carotene using acid–base-induced deep eutectic solvent liquid–liquid microextraction (DES-LLME), liquid–liquid microextraction (LLE), and ultrasound-assisted liquid–liquid microextraction (UA-LLE) from juices of watermelon, grapefruit, tomato, and guava. The extraction efficiency of DES-LLME was compared with other organic solvents such as petroleum ether, acetone, and methanol. To perform the extraction, 600 µL of fatty acid deep eutectic solvent (2C_9_:1C_10_:1C_11_) was mixed with 400 μL of ammonium hydroxide (NH_3_H_2_O) and vortexed for 30 s. The lycopene and β-carotene content of the extract was measured using HPLC. The extraction efficiency obtained was 96% for β-carotene and 90% for lycopene within 8 min of extraction, while liquid–liquid microextraction (LLE) took 30 min for the complete extraction using acetone, methanol, and petroleum ether and had an extraction efficiency of 75–80%. Ultrasound-assisted LLE using methanol had an extraction efficiency of 80–97% after 15 min of extraction. Deep eutectic solvents had higher extraction efficiency compared to other solvents. Martínez et al. [193] studied the extraction of carotenoids from fresh biomass of yeast cells of *Rhodotorula glutinis* using PEF. The yeast biomass was resuspended in a citrate phosphate McIlvaine buffer of pH 7.0 to a final concentration of about 10^8^ cells/mL. This was treated for 150 μs at an electric field of 15 kV/cm and total specific energy of 37.12 kJ/kg. This treatment irreversibly electroporated 90% of the cells. Then, the PEF-treated samples were incubated in ethanol at two conditions, i.e., 24 h at 20 °C and pH of 7.0 and 24 h at 25 °C and pH of 8.0. The yield of carotenoids was 240 μg/g d.w. and 375 μg/g d.w., respectively, at either incubation condition. Cardenas-Toro et al. [194] extracted out carotenoids using Soxhlet, percolation, and PLE from a dried mixture of pressed palm (*Borassus flabellifer* L.) fiber and husk. For Soxhlet extraction, 5 g of sample was refluxed with 100 cm^3^ of ethanol at 78.4 °C and hexane at 69.1 °C for 6 h. For percolation, 2 g of sample was extracted at 35–5 °C using ethanol at a flow rate of 1.6–2.4 g/min. For PLE, 2 g of sample was extracted with ethanol for 5 min at 2–6 MPa and 35–55 °C. Carotenoids obtained were 317 µg/g of extract from ethanolic Soxhlet extraction and 465 µg/g of extract from hexane Soxhlet extraction. The recoveries of carotenoids in percolation and PLE were 246 µg/g and 675 µg/g of extract, respectively. The carotenoid fucoxanthin can be extracted using solid–liquid extraction, SFE, and percolation. Obluchinskaya et al. [195] reported that a NADES solvent consisting of lactic acid and choline chloride in the ratios 3:1 (NADES1) and lactic acid: glucose: water in 5:1:3 (NADES2) can also be used for the extraction of fucoxanthin from brown seaweed *Fucus vesiculosus*. The study concluded that the heating and dilution of these NADES with 30% of water increases the yield of fucoxanthin via NADES. UAE for 60 min at 25 °C was preferred for greater recovery of fucoxanthin. Under these conditions, the recovery (1.0 mg/g DW seaweed) of fucoxanthin using NADES was comparable to the highest recovery (1.10 mg/g DW seaweed) of fucoxanthin that was obtained from maceration with ethanol for 24 h and percolation at a 1:10 *w*/*v* seaweed to solvent ratio at 25 °C. 

### 5.2. Polyphenols

Pavlić et al. [196] studied the NADES extraction of polyphenols from dried wild thyme (*Thymus serpyllum* L.) dust. For the extraction, 0.05 g of sample was mixed with 20 different NADES, each at a sample-to-solvent ratio of 1:20 mL/mL, and the extraction was carried out for 60 min at 50 °C in a water bath placed in a magnetic stirrer hot plate. To further aid the separation of extract from solvent, 4 mL of water was added and centrifuged at 4000 rpm for 15 min. The use of L-proline (Pro)–glycerin (Gly)–water (H_2_O) NADE solvent at a mixture ratio of 1:2:1 with a water content of 5.68% extracted out the highest polyphenols compared to other NADE solvents. The yield of polyphenols was 71.43 mg GAE/g when 1 g of sample was extracted using 28 g Pro-Gly-H_2_O solvent. Popovic et al. [197] studied the green extraction of polyphenols from sour cherry (*Prunus cerasus* L.) pomace using NADES. To perform the extraction, 300 mg of freeze-dried sample was mixed with 4 mL deep eutectic solvent [1:1 M choline chloride (ChCl) as HBA and malic acid, urea, or fructose as HBD] and the extraction was carried out at 50 °C for 45 min with a stirring speed of 650 rpm. The obtained extract had 3238.32 μg/g of total phenols, 2442.93 μg/g of total anthocyanins, 418.00 μg/g of total flavonoids, and 377.39 μg/g of total phenolic acids. Frohlich et al. [198] optimized UAE for the extraction of phytochemicals from dried leaves of clove (*Syzygium aromaticum*) using 99.5% ethanol. It was found in the study that extraction using a solvent-to-sample ratio of 35 mL/g at 70 °C and amplitude of 85% for 25 min gave the highest yield. This resulted in a total extract yield of 14.63%, and the yield of eugenol was 2.94 g/kg of leaves. Domínguez-Rodríguez et al. [199] studied EAE of non-extractable bioactive polyphenol from sweet cherry (*Prunus avium* L.) pomace. In this study, 0.38 g of sweet cherry pomace was extracted using 1 mL methanol at different pH (3–10), temperature (30–70 °C), and enzyme concentrations (1–120 μL/g) for 10–300 min. The optimized conditions were a pH of 10, a temperature of 70 °C, an enzyme concentration of 2 µL/g, and an extraction time of 18.4 min. The recovery of polyphenols at the optimized conditions was 1.1 mg GAE/g sample. Hwang et al. [200] studied the PEF extraction of narirutin and hesperidin from dried *Citrus unshiu* peels. For this, 30 g of the sample was immersed in distilled water and was treated at a 5 kW pulse generator, 50 Hz pulse frequency, and 3 kV/cm electric field for 60 and 120 s at room temperature. The total yield of extract was higher in the sample treated for 120 s, and the yields of hesperidin and narirutin were 46.96 mg/100 g and 8.76 mg/100 g of the sample, respectively. Velásquez et al. [201] designed 10 NADES via lyophilization and used them for the ultrasound-assisted extraction of anthocyanins from *Chilean Luma Chequen* (Molina) A. Gray berry. It was found in the study that the highest recovery of total anthocyanins (3.30 mg/g DW) was obtained for NADESs prepared using lactic acid and glucose in the ratios 8:1, followed by NADESs prepared using choline chloride: glycine (4:6) (3.30 mg/g DW), glycine: glucose (8:1) (3.06 mg/g DW) and tartaric acid: glycine (4:1) (3.03 mg/g). The anthocyanin content of extracts based on NADES was significantly higher than ethanol (1.16 mg/g DW), except for NADESs prepared using tartaric acid: glycine (1:2) (0.81 mg/g DW). Grdiša et al. [138] studied the extraction efficiency of pyrethrins from dried flower heads of Dalmatian pyrethrum (*Tanacetum cinerariifolium/Trevir. Sch. Bip*.) using maceration, UAE, and matrix solid-phase dispersion (MSPD). A sample size of 0.25 g was used in all three extraction methods. Maceration extraction was performed using different solvents, i.e., acetone, ethanol, and ethyl acetate at different volumes, i.e., 5, 7, 9, and 11 mL, at different extraction times, i.e., 0.5, 1, 2, and 3 h, at the stirrer rotational speed of 200, 300, 400, and 500 rpm. UAE was carried out using 5 mL acetone at 50 °C for 60 min at 1200 W and 35 kHz. In MSPD, the sample was mixed with 0.50 g of florisil and 0.40 g of Na_2_SO_4_, after which florisil was activated at 160 °C and washed with n-hexane and methanol. It was then treated with solvents such as acetone and ethyl acetate at 1:1 (*v*/*v*) and extracted using a solid phase extractor. It was found in the study that the highest extraction efficiency of pyrethrin was obtained in maceration (0.62%), followed by MSPD (0.59%) and UAE (0.49%). Sharma et al. [202] optimized MAE for the extraction of phytochemicals such as phenols, flavonoids, ascorbic acid, and tannins from dried fruits of *Ficus racemosa*. The optimized conditions for the extraction were sample to water ratio of 1:15, pH of 3.5, microwave power of 360.55 W, and extraction time of 30 s. These extraction conditions resulted in the extraction of 31.19 mg/100 mL of ascorbic acid, 35.14 mg/100 mL of gallic acid, 14.06 mg/100 mL of tannic acid, 50.86 mg/100 mL of chlorogenic acid, 36.96 mg/100 mL of quercetin. Oroian et al. [203] evaluated the extraction efficiency of flavonoids and polyphenols from crude pollen (collected from a local beekeeper in Suceava County, Romania) using UAE. To perform the extraction, 30 g of pollen sample was mixed with 1 liter of 80% methanol and extraction was carried out at 40.85 °C and 100% amplitude for 14.30 min. The extraction of total phenols and total flavonoids was 366.1 mg GAE/100 g and 592.2 mg QE/g of the sample, respectively. De Queiroz et al. [204] optimized the MAE for the extraction of phenols and tannins from the dried stem bark of *Stryphnodendron adstringens.* The extraction was carried out by adding 0.075 g of sample in 1 mL of water and heating it at 106–134 °C for 0.48–2.12 min. These conditions extracted out 15.91–18.69% tannins and 16.36–22.12% phenols from the studied sample. In a study conducted by Azman et al. [97] on the extraction of free and bound phenolics from dried black currant (*Ribes nigrum* L.) skins, it was found that acetic buffer solvent resulted in the highest free anthocyanin (1712.3 mg/100 g), free hydroxycinnamic acid (268 mg/100 g), total phenolic content (3702 mg GAE/100 g), and DPPH inhibition activity (60.7%) compared to other solvents, i.e., water, methanol and a mixture of methanol and water. The use of acetic acid as a co-solvent with other solvents such as water and ethanol has also been reported to extract the phytochemicals from colored vegetables [205]. Jamaludin et al. [128] optimized the extraction of bioactive compounds from noni fruits using high hydrostatic pressure. This study was carried out in two parts. In the first part, the effect of each extraction parameter (ethanol concentration, pressure, and extraction time) was studied individually on the yield of bioactive compounds (scopoletin, alizarin, and rutin), and in the second part, the combined effect of the extraction parameters was studied on the yield of bioactive compounds using the Box-Behnken Design of RSM. The highest yield of bioactive compounds, i.e., scopoletin (82.4%), alizarin (77.2%), and rutin (82.2%), were found at 544 MPa, with an extraction time of 15 min and ethanol concentration of 65%. The extraction of phenyletanes and phenylpropanoids of Rhodiola rosea L. using NADES was studied by Shikov et al. [206]. The highest concentration of total phenyletanes and phenylpropanoids (26.10 mg/g) was obtained using NADES prepared using L-lactic acid, fructose, and water in the ratios 5:1:11 mL/mol when the particle size of *Rhodiola rosea* L. rhizome was in between 0.5–1 mm and the extraction was carried out for 154 min at 22 °C and extraction modulus of 40. Razboršek et al. [132] performed choline chloride-based UAE NADES extraction of phenolic compounds from chokeberry (*Aronia melanocarpa*) and compared the results with those obtained from 80% methanolic extract. The highest total phenols (36.15 mg GAE/g DW) and total flavonoids (4.71 mg rutin/g DW) were obtained for NADES prepared using choline chloride, fructose, and water in the ratios 2:1:1. This was significantly higher than 80% methanol, i.e., 27.11 mg GAE/g DW for total phenols and 3.37 mg rutin/g DW for flavonoids. The application of methyl acetate under pressurized conditions for the extraction of Crambe seed oil has been reported to have higher phytosterol and tocopherol values compared to the Soxhlet method [207] Castro-López et al. [208] studied polyphenol extraction from pomegranate (*Punica granatum*) peels, walnut (*Juglans regia*) shells, hojasen (*Cassia fistula*) leaves, and moringa (*Moringa oleifera*) leaves using different extraction methods, i.e., maceration, decoction, UAE, and MAE. For maceration, 0.2 g of sample was treated with 10 mL deionized water at a sample-to-solvent ratio of 1:50 at room temperature in a magnetic stirrer for 2 h. For decoction, 0.2 g of sample was treated with 10 mL deionized water at a sample-to-solvent ratio of 1:50 in an oven at 60 °C for 2 h. The UAE was carried out at 25 °C in a sonicated water bath for 60 min using a sample-to-solvent (deionized water) ratio of 1:50. For MAE, deionized water at a sample-to-solvent ratio of 1:50 was used at 550 W and 70 °C for 90 s. Higher polyphenol content was obtained using MAE followed by decoction, UAE, and maceration methods. Total polyphenol yields of 6.4–18.92 mg GAE/g, 1.17–12.8 mg GAE/g, 2.73–15.19 mg GAE/g, and 1.68–12.69 mg GAE/g were obtained for pomegranate peel, walnut shell, moringa leaves, and hojasen leaves, respectively. Jovanovic’ et al. [209] extracted polyphenols from the air-dried aerial part of *Thymus serpyllum* L. using maceration, heat-assisted extraction (HAE), and UAE. Maceration was carried out using ethanol and water solutions containing 30%, 50%, 70%, and 96% ethanol. The particle sizes of the powder used for extraction were 0.3, 0.7, and 1.5 mm, and to perform the extraction, solid-to-solvent ratios of 1:10, 1:20, and 1:30 were used for the extraction times of 5, 15, 30, 60 and 90 min. In HAE, solvent concentrations and sample-to-solvent ratios were the same as that of maceration; however, the extraction was carried out at 80 °C for 5, 15, and 30 min in an incubator shaker. In UAE, solvent type, solid-to-solvent ratio, particle size, and extraction time were similar to those of HAE. The extraction was carried out at 25 °C and 80% amplitude and a 750 W output ultrasonic processor with a 20 kHz converter having a solid titanium probe of 19 mm diameter. The total phenolics extracted using maceration, HAE, and UAE were 19.56 mg GAE/L, 22.60 mg GAE/L, and 24.94 mg GAE/L, respectively. Porto and Natalino [210] studied the SFE of polyphenols from dried white grape Marc (*Vitis vinifera*) seeds. They used 100 g of sample in an SFE pilot plant (SCF100 series 3 PLC-GR-DLMP, Separeco S.R.L, Pinerolo, Italy) equipped with a 1 L extraction vessel, and the extraction was carried out for 13 min at a pressure of 80 bar, a temperature of 40 °C, and a CO_2_ flow rate of 6 kg/h along with 57% *v*/*v* of ethanol–water (20% *w*/*w*) mixture as co-solvent. The total polyphenol yield in this extraction was 7132 mg GAE/100 g DM. The extraction of phlorotannins from brown algae using NADES is reported by Obluchinskaya et al. [211]. The study reported that the use of aqueous NADES solutions (50–70%) based on choline chloride with added lactic or malic acid and betaine and malic acid gave a 6—72% yield of phlorotannins. Sharif and Bennet [212] compared maceration and reflux methods for the extraction of polyphenols from freeze-dried ginger rhizomes using various solvents viz. ethanol, methanol, and acetone. For maceration, 10 g of sample was used with 300 mL of solvent and placed in an orbital shaker for 8 h. In the reflux extraction, a 2.5 g sample was extracted with 50 mL solvent at 90 °C for 30 min. The total phenol contents obtained using ethanol for the maceration and reflux extraction were 263 and 205.4 mg/100 g GAE, respectively. In the case of acetone, the total phenols yield was 216 mg/100 g GAE for maceration and 184 mg/100 g GAE for reflux extraction, while when using methanol it was 148 mg/100 g GAE for maceration and 95 mg/100 g GAE for reflux. This shows the lower extraction efficiency of reflux extraction compared to maceration. Altuner et al. [180] studied the HPP extraction of phenolic compounds from *Maclura pomifera* fruits using two solvents, i.e., methanol and solvent cocktail (distilled water:ethanol:methanol:acetone:dichloromethane—1:2.5:2.5:2:2) at two different pressures, i.e., 250 and 500 MPa for 10 min. The highest recovery of the phenolic compounds (913.173 µg GAE/mL) was found at 500 MPa using the solvent cocktail. The yield of total phenolics in HHP extraction was higher than in Soxhlet extraction (316.877 µg GAE/mL). Moreira et al. [129] studied the effect of HHP on biological activities and phenolics composition of winter savory leaf extracts and reported that the extract obtained at 348 MPa using 35% (*v*/*v*) ethanol had the highest concentration of individual phenolic compounds and a minimum bactericidal concentration of 20 mg/mL against *Listeria monocytogenes*. The HHP extract was also found to induce less oxidative damage than the control extract. 

### 5.3. Phytosterols

De Aquino et al. [213] studied the effect of thermal pre-treatment on the enzyme (protease)-assisted aqueous extraction and yield of phytosterols from sunflower (*Helianthus annuus* L.) seeds. The thermal pre-treatment was performed by immersing 150 g of whole seeds in distilled water at room temperature in the ratio of 1:3 (*w*/*v*). Excess water was removed after 3 h and the sample was placed in an oven with air circulation (Marconi, Model MA035) at 120 °C for 60 min. A commercial enzymatic preparation, i.e., Alcalase^®^ 2.4 L FG was used as a source of protease enzyme. The extraction for both thermally pre-treated and untreated samples was carried out for 3 h at 40 °C, pH of 8, and enzyme concentration of 9% *v*/*v*. The total yield of the oil using this method was 15.59%. The total yield of phytosterols in the oil extracted without thermal treatment was 149.41 mg/100 g oil, and the major phytosterols were campesterol (15.80 mg/100 g), stigmasterol (21.46 mg/100 g), γ-sitosterol (12.07 mg/100 g), and β-sitosterol (100.07 mg/100 g). The yield of total phytosterols from the thermally pre-treated samples was 133.66 mg/100 g. The major phytosterols obtained were campesterol (15.99 mg/100 g), stigmasterol (18.88 mg/100 g), γ-sitosterol (8.72 mg/100 g), and β-sitosterol (90.08 mg/100 g). Hien and Minh [214] compared UAE and enzyme-assisted UAE for the extraction of oil and phytosterols from dried pumpkin (*Cucurbita pepo* L) seeds. The extraction was carried out using hexane for 4.5 h at a sample-to-solvent ratio of 1:6, frequency of 40 kHz, and temperature of 60 °C. In enzyme-assisted UAE, commercial enzyme Alcalase^®^ 2.4 L FG with enzyme activity of 3.9 U/mL was used along with the above parameters. The oil extraction yield was 95.46% in UAE, while 91.87% was obtained from enzyme-assisted UAE. The phytosterol content was 2017.5 mg/100 mL in UAE-extracted oil and 2327.7 mg/100 mL in oil extracted from enzyme-assisted UAE. UAE was found to be effective for oil extraction, while the phytosterol extraction was more efficient with the enzyme-assisted UAE. Jalani et al. [215] extracted phytosterols from sludge palm oil (also known as palm acid oil) and empty fruit bunch (*Elaeis guineensis*) residual oil. In this study, a 5 g sample was extracted at 90 °C for 1 h with 50 mL of ethanol at the sample-to-solvent ratio of 1:10. The quantity of the phytosterols in the sludge palm oil was 500 ppm and the content of phytosterols in unsaponifiable form was 6.19%. In empty fruit bunch residual oil, phytosterol content was 450 ppm and the phytosterols in unsaponifiable form were 4.58%. Jafarian Asl et al. [216] compared Soxhlet and SFE extraction of phytosterols from rapeseed (*Brassica napus* L.) oil. Soxhlet extraction was carried out using a 15 mg sample in 150 mL ethanol at the sample-to-solvent ratio of 1:10 at different temperatures (40, 60, and 80 °C) for 1 h. SFE was carried out for 1 h with CO_2_ and co-solvent ethanol having flow rates of 5 mL/min and 0.5 mL/min, respectively. This extraction was carried out at different pressures ranging from 100–400 bar. The highest yield (87%) of phytosterols was obtained in Soxhlet extraction at 40 °C and SFE at 350 bar. The lowest phytosterol yield (21%) was obtained with SFE at 100 bar. Ibrahim et al. [217] studied the MAE extraction of β-sitosterol from cocoa (*Theobroma cacao*) shell waste. The extraction was carried out using a 100 g sample with 300 mL, 99% ethanol in the sample to solvent ratio of 1:3 at 500 W and 70 °C for 10 min. The total extract obtained was 13% based on the one-factor-at-a-time (OFAT) approach and the sitosterol present was 3546.1 mg/100 g extract. Regalado-Rentería et al. [218] extracted phytosterols (β-sitosterol and γ-tocopherol) using maceration–percolation (MP) from prickly pear (*Opuntia ficus* L.) seeds. For the percolation, 100 g crushed seeds were placed inside the percolation column with 400 mL, 95% n-hexane at 50 °C for 24 h. This method yielded 15.54% of oil. The yields of β-sitosterol and γ-tocopherol in the oil were 58.3 mg/100 g and 23.8 mg/100 g, respectively. 

### 5.4. Saponins

Li et al. [219] studied the UAE of saponins from powdered *Aralia taibaiensis* root bark. The extraction was carried out in a water-bath sonicator with different ethanolic concentrations (50, 60, 70, 80, and 90%), time durations (10, 20, 30, 40, and 50 min), temperatures (40, 50, 60, 70, and 80 °C), sample to solvent ratios (5, 10, 15, 20, and 25 g/mL), ultrasound power (100, 200, 300, 400, and 500 W) and number of extractions (1, 2, 3, 4, and 5). The highest total saponin (11.45%) content was obtained when 5 g of sample was extracted with 75 mL of 73% ethanol at 400 W and 61 °C for 34 min. Liu et al. [220] studied the EAE of saponins from powdered *Acanthopanax senticosus*. The extraction was carried out at different enzyme concentrations (1000, 5000, and 9000 U/g), time durations (45, 55, and 65 min), temperatures (40, 50, and 60°C), and solvent pH values (5.4, 6, and 6.6). The highest extraction yield of saponins (17.8 mg/g sample), was obtained when 2 g of sample was extracted with 6963 U/g of enzyme mixture (cellulase and pectinase at a ratio of 2:3) at pH 6 and a temperature of 53.7 °C for 60 min. Yang et al. [221] studied the extraction of four bioactive steroidal saponins (protodioscin, protogracillin, pseudoprotodioscin, and pseudoprotogracillin) from the dried rhizome of *Dioscorea nipponica,* also known as Dioscoreae Nipponicae Rhizoma (DNR), using NADES. A mixture containing ChCl and malonic acid in a molar ratio of 1:1 with 30% water was used as the NADE solvent. For a sample weighing 50 mg, the optimal extraction conditions were 1 mL NADE solvent, an extraction time of 23.5 min, a liquid–solid ratio of 57.5 mL/g, a water content of 54%, and ultrasonic conditions of 300 W and 40 kHz. The recovery yield of four steroidal saponins was between 98.8 and 107.5% compared to standard steroidal saponins. The extract consisted of 64.99 mg/g of total saponins where 29.39 mg/g was protodioscin, 15.86 mg/g was protogracillin, 9.71 mg/g was pseudoprotodioscin, and 3.66 mg/g was pseudoprotogracillin. Ramli et al. [222] studied the MAE of saponins from dried furcraea (*Furcraea selloa var. marginata*) leaves using water, ethyl acetate, and ethanol. The extraction was carried out using a 3 g sample in 200 mL solvent at a ratio of 1:24, frequency of 2.45 GHz, and power of 1000 W at 90 °C for 9 min. The extraction yields obtained from aqueous, ethyl acetate, and ethanolic extract were 5.77%, 8.07%, and 6.67%, respectively. The saponin contents in the samples extracted using water, ethyl acetate, and ethanol were 0.0514 g/mL, 0.0453 g/mL, and 0.0344 g/mL extract, respectively. Wu et al. [223] studied the extraction of oil and tea saponins from dried *Camellia oleifera* kernel powder using subcritical water extraction and Soxhlet extraction. For subcritical water extraction, a 20 g sample was dissolved in water at the sample-to-solvent ratio of 1:3 to 1:25 with an agitation rate of 150 rpm, a temperature of 60–160 °C, and pressure of 2–7 MPa for 5–60 min. For Soxhlet extraction, a 5 g sample was extracted with 100 mL petroleum ether in a Soxhlet extractor at 65 °C for 6 h. Petroleum ether was evaporated and oil was obtained. From subcritical water extraction, the highest yield of oil (94.07%) was obtained at the solvent: sample concentration of 10.79 mL/g, a temperature of 133.59 °C, and extraction time of 32.03 min. The yield of saponins in the oil sample was 71.38%. From Soxhlet extraction, an oil yield of 91.27% was obtained when extraction was carried out at 121.11 °C for 32.07 min with a solvent concentration of 8.33 mL/g. The total saponin yield was 74.21% from the oil sample. Gong et al. [224] optimized the recycling reflux extraction process for the extraction of saponins [total saponins, ginsenoside (Rg1), notoginsenoside (R1), ginsenoside (Rb1), and ginsenoside (Rd)] from *Pranax notoginseng* using a Box–Behnken design. The extraction was carried out by adding 50 g of sample and 500 mL of the ethanol–water mixture (70–90%) in the extraction tank (heat constant temperature tanks), soaking for 2 h, pumping the extract to the concentration tank, evaporating the solvent, collecting the condensed solvent in a storage tank, and pumping it back to the extraction tank with a fixed flow rate. The flow rate was set to the values required by the experimental designs. The variables of the extraction were ethanol concentrations (70–90% *v*/*v*), extraction times (4–10 min), and the ratio of recycling ethanol rate (0–0.04/min). An increase in saponin yield was observed with an increase in ethanol concentration at first (up to 32.55% at 80% ethanol), after which it decreased (25.89–32.03% at 90% ethanol). The total saponin purity was also increased with an increase in ethanol concentration. The yield of ginsenosides Rb1 and Rd was also increased with an increase in extraction time. The conditions optimized for the extraction of saponins from this study were 79–82% ethanol, 6.1–7.1 h of extraction time, and 0.039–0.040 min^−1^ ratio of recycling ethanol rate. The concentrations of ginsenoside (Rg1), notoginsenoside (R1), ginsenoside (Rb1), and ginsenoside (Rd) observed in the sample at the optimized extraction conditions were 14.91 mg/g, 48.14 mg/g, 50.71 mg/g, and 10.45 mg/g, respectively. 

### 5.5. Isoprenoids

Lanjekar and Rathod [225] optimized the extraction of glycyrrhizic acid from *Glycyrrhiza glabra* (Liquorice root) powder using choline chloride (ChCl): lactic acid (1:1), ChCl: dextrose (2:1), ChCl: glycerol (min1:1), ChCl: malic acid (1:1), ChCl: citric acid (1:1), ChCl: oxalic acid (1:1), and ChCl: succinic acid (1:1) as different natural deep eutectic solvents. In this extraction, 2 g licorice powder having a moisture content of 7.78% was treated with 20 mL NADES in an overhead stirrer for 60 min at 400 rpm and 30 °C. The mixture was then centrifuged at 8000 rpm for 10 min and analyzed using HPLC. The highest glycyrrhizic acid yield was 43.65 mg/g of the sample using ChCl: succinic acid as solvent. The yield of glycyrrhizic acid was 42.82 mg/g using ChCl: lactic acid as solvent, 23.25 mg/g using ChCl: dextrose, 14.37 mg/g using ChCl: glycerol, 30.67 mg/g using ChCl: citric acid, 36.70 mg/g using ChCl: malic acid, and 39.60 mg/g using ChCl: oxalic acid. The extraction of glycyrrhizic acid from liquorice roots using NADES has also been reported by Shikov et al. [226]. The yields of glycyrrhizic acid in NADES based on sucrose and lactic acid (3:1), sorbitol and lactic acid (3:1), and choline chloride and lactic acid (1:3) were higher (38–60 mg/g) than its yield in water (<30 mg/g). Rodrigues et al. [227] extracted triterpenoids from dried leaves of *Acacia dealbata* using SFE and Soxhlet extraction. The sample was dried using a forced convection oven at 35 °C for 72 h to a moisture content of 4.5% wt. For Soxhlet extraction, different solvents such as 99.5% ethanol, 99% hexane, 99% ethyl acetate, and 99% dichloromethane were used. In this study, 3 g of sample was used with 180 mL solvent for 6 h at 39–78 °C. For SFE, CO_2,_ CO_2_: ethanol (95:5 wt.%), and CO_2_:ethyl acetate (95:5 wt.%) were used as solvents. For extraction, a 25 g sample was loaded into the extraction chamber of a lab-scale Speed Helix SFE System at a flow rate of 12 g/min for 6 h at 40–80 °C. From Soxhlet extraction, the highest total extraction yield obtained was 11.58% using ethanol as solvent. The highest triterpenoid yield obtained was 8201 mg/kg of extract using ethyl acetate as a solvent and 6259 mg/kg of extract using ethanol as a solvent. From SFE, the highest yield was 1.76% using CO_2_ as solvent. The highest triterpenoid yield obtained was 4719 mg/kg of extract using CO_2_: ethanol as solvent and a triterpenoid yield of 4366 mg/kg of extract using CO_2_:ethyl acetate as solvent. Grdiša et al. [138] studied the extraction efficiency of pyrethrins from dried flower heads of Dalmatian pyrethrum (*Tanacetum cinerariifolium/Trevir./Sch. Bip*.) using maceration, UAE, and matrix solid-phase dispersion (MSPD). A sample size of 0.25 g was used in all three extraction methods. Maceration extraction was performed using different solvents, i.e., acetone, ethanol, and ethyl acetate at different volumes, i.e., 5, 7, 9, and 11 mL, at different extraction times, i.e., 0.5, 1, 2, and 3 h at stirrer rotational speeds of 200, 300, 400, and 500 rpm. UAE was carried out using 5 mL acetone at 50 °C for 60 min at 1200 W and 35 kHz. In MSPD, the sample was mixed with 0.50 g of florisil and 0.40 g of Na_2_SO_4_, (florisil was activated at 160 °C and washed with n-hexane and methanol). It was then treated with solvents such as acetone and ethyl acetate at 1:1 (*v*/*v*) and extracted using a solid phase extractor. It was found in the study that the highest extraction efficiency of pyrethrin was obtained in maceration (0.62%), followed by MSPD (0.59%) and UAE (0.49%). Yingngam et al. [228] optimized the MAE of pentacyclic triterpenes from dried Gotu kola (*Centella asiatica* L.) leaf powder. Extraction was carried out with ethanol in a modified microwave oven using a 5 g sample at a frequency of 2.45 GHz. Various parameters used for extraction were ethanol concentrations (0, 25, 50, 75, and 100%), solvent-to-sample ratios (10:1, 20:1, 30:1, 50:1, and 70:1 mL/g), microwave powers (300, 450, 600, 700, and 800 W) and extraction times (0.5, 1, 2.5, 5, 7.5 and 10 min). The highest pentacyclic triterpene extracted was 36.19 mg/g of the sample when the extraction was carried out for 2.75 min at 300 W with 50% ethanol at a sample-to-solvent ratio of 1:10. Zheng et al. [229] optimized the UAE of triterpenoids from dried medicinal mushroom *Ganoderma lucidum* (Fr.) Krast powder. Extraction was carried out in a 3 L ultrasonic cleaning bath using 1 g sample and 40 mL 95% ethanol with different ultrasonic powers (140, 175, and 210 W), extraction temperatures (70, 80, and 90 °C), solvent to sample ratios (30, 40, and 50 mL/g) and time intervals (60, 90, and 120 min). The extracted sample was centrifuged at 8000× *g* for 10 min. The highest triterpenoid yield (0.48%) was obtained when extraction was carried out with a solvent-to-sample ratio of 50 mL/g at 210 W and 80 °C for 90 min. Fernandez-Pastor et al. [230] optimized the extraction of triterpene acids (oleaonic acid and maslinic acid) from dried olive (*Olea europaea* L.) skin powder using MAE and Soxhlet extraction. For MAE, 0.25 g, 0.5 g, and 1 g samples were mixed with ethyl acetate or methanol as a solvent with varying sample-to-solvent ratios (1:10, 1:20, and 1:40) at 85 °C for 10 min. Samples were then dried at 80 °C for 8 h using a forced air oven. For Soxhlet extraction, 3.75, 7.5, and 15 g samples were mixed with ethyl acetate or methanol at varying sample-to-solvent ratios (1:10, 1:20, and 1:40) at 65–70 °C for 30 and 60 min. From MAE, the highest extraction yield obtained was 180.3 mg/g of the sample using methanol as solvent at a 1:10 sample solvent ratio for the extraction period of 2 min. The oleaonic acid obtained was 11.53 mg/g of sample and the maslinic acid obtained was 15.22 mg/g of sample. From Soxhlet extraction, the highest extraction yield obtained was 179.7 mg/g of the sample using methanol as solvent at a 1:10 ratio for 30 min. The oleaonic acid obtained was 6.55 mg/g of sample and the maslinic acid obtained was 11.79 mg/g of sample.

### 5.6. Polysaccharides and Dietary Fiber

Gong et al. [231] studied the SFE of polysaccharides from dried fallen *Ginkgo biloba* leaf powder. For the extraction, a 20 g sample was placed in a supercritical extraction device and the extraction was carried out using SC-CO_2_ and 15% co-solvent having strengths of 60, 70, and 80% and at the temperatures of 50, 60, and 70 °C, pressures of 35, 40, and 45 MPa, and time intervals of 80, 100, and 120 min. The extract was centrifuged at 6000 rpm for 15 min and analyzed. The highest yield (10.13 g/100 g) of polysaccharides was obtained with 68% co-solvent at the extraction conditions of 42 MPa, 63°C, and 99 min. García et al. [232] studied pressurized liquid extraction of total dietary fiber from dried pomegranate (*Punica granatum* L.) peel and fruit. For the extraction, 3.75 g of powdered sample was mixed with 11.25 g of sand and the extraction was carried out in a pressurized liquid extractor at 1500 psi for 20 min. The total dietary fiber extracted was analyzed using an enzymatic gravimetric method and a yield of 30% was obtained from the peel and 18% from the fruit. Hussain et al. [233] optimized the UAE of soluble dietary fiber from dried sea buckthorn (*Hippophae rhamnoides* L.) pomace powder. Samples were pretreated by mixing with 0.1% citric acid at the sample-to-acid ratio of 1:25 and incubated in a water bath at 80 °C for 1 h. Extraction was carried out using a Digital Sonifier^®^ S450 CE (Richmond Newtown, VA, USA) attached with a 13 mm diameter disruptor horn. The extraction was carried out at different sonication temperatures (60, 70, and 80 °C), powers (100, 130 and 160 W), and time intervals (30, 45, and 60 min). The extract so obtained was further centrifuged at 6000 rpm for 10 min to obtain the dietary fiber. The highest extraction yield obtained was 17.82% under the conditions of 130 W and 70 °C for 45 min. Rivas et al. [234] optimized the SFE of dietary fiber from dried pomegranate (*Punica granatum* L.) peel. The extraction was carried out using 40 g powdered sample inside a 100 mL stainless steel column with a CO_2_ flowrate of 2 L/min and different extraction conditions such as pressure (250, 275, and 300 bar), temperature (45, 50, and 55 °C), and time (2, 3, and 4 h). Dietary fiber was estimated using the alcohol–insoluble residue method. The highest dietary fiber obtained was 49.37 g/100 g of the sample under the extraction conditions of 45 °C and 300 bar for a duration of 2.2 h. Douard et al. [235] optimized NADES extraction of cellulose nanocrystals from cotton sheets obtained from the paper industry. The extraction was carried out using a 2 mg cotton sheet with a mixture of 63 g oxalic acid and 69.8 g ChCl as NADE solvent under differing conditions such as cellulose concentrations (1, 1.5, and 2%), temperature (60, 75.5, and 95 °C), and time intervals (2, 9, and 16 h). After extraction, cellulose was washed and filtered out through a 1 µm membrane using 200 mL of deionized water, and the filtrate was centrifuged at 10,000 rpm for 15 min. The highest yield of cellulose nanocrystals (35.5%) with a crystallinity index of 80% was obtained at 95 °C for 6 h with a 2% cellulose concentration. Gan et al. [236] studied the extraction of soluble dietary fiber from dried grapefruit (*Citrus paradisi*) peel powder using microwave–enzymatic treatment (MET), microwave–sodium hydroxide treatment (MST), and microwave–ultrasonic treatment (MUT). Microwave treatment was carried out using a 3 g sample in each of eight polyfluoroalkoxy tubes that were treated using a microwave cracker at 500 W and 80 °C for 40 min. For MST, a 5 g microwave-treated sample was mixed with 1% NaOH using a magnetic stirrer in a water bath at 200 rpm and 50 °C for 30 min and then centrifuged at 4800 rpm for 10 min. For MET, a 5 g microwave-treated sample was mixed with 240 mg cellulase (3000 U/g) at a pH of 4.5 and 1% heat stable α-amylase at pH 5, then incubated in a water bath for 30 min at 90 °C. When the temperature reached 60 °C, the pH was maintained at 6, and 0.05% papain was added and incubated at this temperature for 30 min. For MUT, a 5 g microwave-treated sample was placed in a Sonicator JY92-Ⅱ (Ningbo Scientz. Biotechnology Co. LTD, Ningbo, China) at 200 W and 25 °C for 10 min. The yields of the dietary fiber from MST, MET, and MUT were 17.19 g/100 g, 9.13 g/100 g, and 8.35 g/100 g samples, respectively. Cheikh Rouhou et al. [237] compared different solvents for the extraction of dietary fiber from ground cactus (*Opuntia ficus indica*) rackets. Water and ethanol were used as a solvent in maceration extraction and lemon juice as a solvent in steam extraction. For maceration using water, hot water was used for extraction at the sample-to-solvent ratio of 1:30 at 100 °C for 30 min and 1 h. For maceration using ethanol, 80% ethanol was used at the sample-to-solvent ratio of 1:10 for 30 min and 1 h at room temperature. For steam extraction using lemon juice, a sample-to-solvent ratio of 1:30 at 220 °C and 2 bar pressure at pH 2 were used for 30 min and 1 h. The highest fiber content (86.66%) was obtained in lemon juice steam extraction followed by maceration with water (85.81%) and ethanol (84.88%) after 1 h of extraction. Zheng et al. [229] optimized polysaccharide extraction from dried medicinal mushroom *Ganoderma lucidum* (Fr.) Krast powder. The maximum polysaccharide yield of 0.72% was obtained after 90 min of extraction at a solvent-to-sample ratio of 40 mL/g, power of 175 W, and temperature of 80 °C. The yield of polysaccharides was 0.68%. Gómez et al. [238] studied microwave-assisted natural deep eutectic solvent (MA-NADES) extraction of soluble sugars from banana puree. Different NADES such as malic acid: b-alanine (MA:BA), malic acid: choline chloride (MA:CC), citric acid: choline chloride (CA:CC), and citric acid: b-alanine (CA:BA) were used. The extraction was carried out using a 3 g sample and 30 g NADE solvent at 2.45 GHz and 900 W at varying temperatures of 25, 50, and 70 °C. Water was used along with NADES at the rate of 30, 40, and 50 mL/g and extraction times of 5, 15, and 30 min. The highest soluble sugar yield (106.9 g/100 g) was obtained using 30 mL of water per gram of NADES (MA:BA) at extraction temperatures of 25 °C and extraction time of 30 min. A yield of 86.6 g/100 g was obtained using MA:CC at 70 °C and an extraction time of 30 min using 30 mL water per g of NADES. The extraction using CA:BA yielded 86.1 g/100 g sugar at extraction conditions of 50 °C, water: NADES concentration of 50 mL/g, and the extraction duration of 30 min. The soluble sugar yield was 101.5 g/100 g when extraction was carried out for 30 min at 70 °C using CA:CC containing 30 mL/g water. Liu et al. [239] extracted polysaccharides from dried *Dendrobium nobile* Lindl. stems using sub-critical water extraction. The powdered sample was mixed with water at the sample to solvent ratio of 1:25 inside a vessel and it was purged with analytical-grade inert nitrogen. The extraction was carried out at different temperatures (120, 140, and 160 °C), pressure (0.5, 1, and 1.5 MPa), and time intervals (5, 12.5, and 20 min). The mixture was agitated at 300 rpm in a magnetic stirrer under the above conditions. The obtained extract was concentrated in a vacuum rotary evaporator at 65 °C and centrifuged at 4000 rpm for 10 min. The highest polysaccharide yield (21.88%) was obtained under the optimal extraction conditions of 129.83 °C, 1.12 MPa, and 16.71 min. Ennaifer et al. [108] extracted water-soluble polysaccharides from dried whole *Pelargonium graveolens* plant using decoction. Water was used as solvent at the sample-to-solvent ratio of 1:10, the temperature of 78–98 °C, and extraction times of 8–20 min. The yield of water-soluble polysaccharides at this condition was 6.43%. 

## 6. Conclusions

Phytochemicals are nutritional or non-nutritional bioactive plant compounds found in fruits, vegetables, cereals, and other plant foods. They may have health advantages in addition to basic nutrition, such as lowering the risk of major chronic diseases. The major phytochemicals in plants are carotenoids, polyphenols, isoprenoids, phytosterols, saponins, dietary fibers, and polysaccharides. These phytochemicals can be extracted using conventional methods such as maceration, percolation, decoction, reflux extraction, and Soxhlet extraction, or novel extraction methods including PLE, MAE, UAE, PEF, HHP, LGS, EAE, SFE, and NADES. The suitability of extraction methods depends on many factors; however, it can be broadly concluded that maceration is a good and non-expensive extraction method for heat-labile compounds such as polyphenols, PLE results in better carotenoid extraction, UAE coupled with enzymatic treatment results in increased phytosterol extraction, and NADES results in better phytochemical yield compared to other extraction methods. Maceration is a time-consuming process; hence, SFE, UAE, and PEF can be used as its alternative. However, the higher instrumentation and operational costs limit their application. The Soxhlet extraction method is also a reliable method for the extraction of fat-soluble phytochemicals, but it is unsuitable for samples with high moisture content. More comparative studies are required to obtain a better understanding of suitable extraction techniques. Future studies should be focused on the comparison of extraction techniques working on similar principles. Such studies will provide a clearer and more meaningful comparison of the techniques and solvents used for the extraction of various phytochemicals. 

## Figures and Tables

**Table 1:** Major phytochemicals, their sources, active sites, and the related health benefits.

Phytochemical	Sources	Active Site	Health Benefits	References
**Carotenoids**				
α-carotene	Mango, pear, peach, pumpkin, butternut squash, green bean, okra, avocado, chard, collard greens, tangerine, banana	Pulp of mango, tangerine, avocado, butternut squash, and pumpkin; the green part of okra, chard, collard greens	Regulates gene transcription, protects against lung and prostate cancer, good for eye health	[7,11,12]
β-carotene	Red pepper, carrot, spinach, peaches, brussel sprout, grapefruit, sour cherries, papaya, mango, romaine lettuce	Green parts of plants, flowers, roots, and stems of plants; pulp of mango, grapefruit, papaya, etc.	Enhancement of gap junction communication, enhances immunity	[6,13,14]
Lutein	Asparagus, spinach, kale, green beans, orange pepper, lettuce, broccoli, parsley, pistachio nuts	Leaves of spinach, lettuce, parsley; flower part of broccoli; essential oil of pepper; middle lamella of nuts	Improves immunity, good for eye health	[15]
Lycopene	Tomato, sweet potato, pink grapefruit, pink guava, watermelon, apricot, papaya, rosehip	Skin and pulp of tomato, grapefruit, watermelon, apricot, guava	Improves eyesight, reduces pain, and strengthens bones	[16,17]
Xanthophylls	Pumpkin, papaya, pepper, mushroom	Young leaves of papaya, pumpkin; essential oil of pepper	Antioxidant properties, boosts eye health and blood flow	[18,19]
Cryptoxanthin	Apricot, papaya, peach, cashew apples, seabuckthorn, mandarin, tangerine, lemon	Skin and pulp of cashew apple and citrus fruits	Maintains pulmonary health, prevents arthritis and inflammation; improves immune response	[20,21]
Fucoxanthin	Brown seaweeds, Bacillariophyta, Chromophyta, Macroalgae, Microalgae	Chloroplasts of brown seaweeds	Antioxidant, anti-inflammatory, antihypertensive, anticancerous, antidiabetic, antiobesity and radioprotective properties	[10,22]
**Polyphenols**				
Flavones	Parsley, oregano, rosemary, green olive, pumpkin, watermelon, bell pepper, honey, fava beans, chickpea, field pea	Essential oils of spices, pulp of watermelon and pumpkin	Action against free radicals, protective effects against cardiovascular diseases, cancers, and other age-related diseases	[23,24]
Flavanones	Grapefruit, pumelo, mandarin, lemon	Pulp of citrus fruits	Protective effects against cardiovascular diseases, prevention of inflammation and allergies	[25,26]
Flavanols	Chocolate, tea, grapes	Green and black tea leaves	Action against free radicals, prevention of inflammation and allergies	[27,28]
Anthocyanidins and anthocyanins	Blueberry, cranberry, pomegranate, red grapes, black soybean, purple corn, red cabbage, raspberry	Flesh of berries, skin of grapes, corn fiber	Protective effects against cardiovascular diseases, prevention of inflammation and allergies	[26,29]
Polyphenol amides	Oats, chili, pepper	Capsaicinoids in chili pepper, avenanthramides in oats	Prevention of inflammation and allergies	[23,30]
**Isoprenoids**				
Limonene	Lemon, lime, orange	Oil of orange	Anti-inflammatory, antioxidant, and anti-stress properties, as well as a neuroprotective role in Alzheimer’s disease	[31,32,33]
Myrcene	Mango, guava, thyme, parsley, bay leaves, lemongrass, cardamom, sweet basil, juniper	Essential oil extract of lemongrass, juniper, cardamom	Anxiolytic, antioxidant, anti-aging, anti-inflammatory, and analgesic properties	[34]
Pinene	Cannabis, turpentine tree, ironwort, sage plant	Oil of cannabis, ironwort, and sage plants	Antibacterial, antitumor, anti-inflammatory, and sedative properties	[35,36]
**Phytosterols**				
Campesterol	Banana, pomegranate, pepper, coffee, grapefruit, cucumber, onion, oat, potato, lemongrass	Pulp of bananas, pomegranate, grapefruit; essential oil of pepper, lemongrass, etc.	Used in the treatment of allergy, asthma, psoriasis, rheumatoid arthritis, chronic fatigue syndrome, migraine, and menstrual disorders	[37,38,39]
Sitosterol	Avocado, hazelnut, walnut, soybean, olive, canola	Oil of hazelnut, walnut, olive, canola, soybean	Used in the treatment of an enlarged bladder; reduces the risk of cardiovascular disease, promotes anti-cancer properties	[28,37,38]
Stigmasterol	Soybean, calabar bean, and rapeseed	Oil of soybean, calabar bean, and rapeseed	Has a protective effect against gastric and duodenal ulcers, neurological disorders	[37,38,40]
Campestanol	Soybean, olive, hazelnut, flax, cashew	Oil of soybean, olive, hazelnut, flax, and cashew	Prostate health, hair growth, reduce LDL cholesterol	[37,38,41]
Sitostanol	Pepper, banana, pomegranate, soybean, olive	Oil of pepper, soybean, and olive; pulps of banana and pomegranate	Reduces chance of heart attack and stroke, improves hair growth	[37,38,42]
Stigmastanol	Hazelnut, olive, corn	Oil of hazelnut and olive, as well as corn fiber	Reduces chance of heart attack and stroke, antioxidant activity	[37,38,43]
**Saponins**				
Dammarane	Black gram, garden pea, pigeon pea	Middle lamella of peas and legumes	Exhibits hypoglycemic, virucidal, and antifungal activity	[44,45]
Tirucallane	Sunflower, almond, walnut	Oil of almond, sunflower, and walnut	Has an effect on the transverse tubular system and sarcoplasmic reticulum at lower concentration (10µg/mL), has an effect on skin inflammation and diarrhea	[46,47]
Oleanane	Common bean, black gram, almond	Middle lamella of legumes and oil of almond	Antimicrobial and hypolipidemic activities; aids in the treatment of chronic diseases	[48]
**Dietary fiber**				
Pectin	Apples, apricots, cherries, oranges, carrots, citrus fruits, rose hip	Peels of citrus fruits, middle lamella of cell walls of fruits	Lowers LDL cholesterol; cures diarrhea; promotes the generation of peripheral regulatory T cells	[49,50,51]
Cellulose	Rice, wheat, sisal, jute, hemp, corn, flasks	Rice husk, wheat straw, kernels of corn	Improves insulin sensitivity, gut microbial viability and diversity; reduces the level of bad cholesterol; reduces free radical damage to cells	[52,53,54]
Lignin	Flaxseeds, parsley, carrots, horseradish), Wheat, tomatoes, berries, broccoli, cabbage, green beans, peaches, peas, Brazil nuts, apples	Seeds of tomatoes and berries, stems of cabbage and broccoli, bran of wheat	Lowers the risk of cancer, reduces hot flashes in postmenopausal women, protects from cardiovascular diseases	[55,56,57]
Hemicelluloses	Rice, wheat, nuts, legumes, whole grains	Bran of rice and wheat, middle lamella of legumes, nuts	Improves metabolites from gut microflora; reduces cardiovascular risk	[58,59]
**Polysaccharides**				
Amylose	Corn, rice, quinoa, potato, oats, arrowroot	Starchy endosperm of corn, rice, potato, and oats; powder of arrowroot	Cures immunodeficiency, cancer, inflammation, hypertension, hyperlipidemia	[60]
Amylopectin	White potato, rice, oats, corn	Starchy endosperm of rice, white potato, oats, and corn	Improves intestinal health and increases gut microbiota	[61,60]
Resistant starch	Buckwheat, oats, lentils, peas, beans	Starchy endosperm of oats, buckwheat, and lentils	Cures hypercholesterolemia and obesity; improves gut microbiota	[62]
Arabinoxylan	Rice, barley, guar gum, wheat, finger millet	Starchy endosperm of rice, barley, wheat, and finger millet	Improves gastrointestinal health; reduces diabetics, cancer, and obesity	[63,64,65]

**Table 2:** The optimized conditions for the extraction of phytochemicals using various extraction methods.

Extraction Method	Solvent	Temperature	Pressure	Time Consumed	References
Maceration	Water, aqueous and non-aqueous solvent	Room temperature or cold method (4–15 °C)	Atmospheric pressure	3–7 days or up to months	[103,104,105]
Percolation	Water, aqueous and non-aqueous solvent	Room temperature or under heat (35–70 °C)	Atmospheric pressure	2–24 h	[106,107]
Decoction	Water	Atmospheric pressure	1–2 h	65–70 °C	[108,109]
Reflux extraction	Water, aqueous and non-aqueous solvent	60–100 °C	Atmospheric pressure	15 min–2 h	[110,111,112]
Soxhlet extraction	Organic solvents	65–100 °C	Atmospheric pressure	6–24 h	[113,114]
Pressurized liquid extraction	Water, aqueous and non-aqueous solvent	50–200 °C	50–300 psi	5–20 min	[115,116,117]
Microwave-assisted extraction	Water, aqueous and non-aqueous solvent	40–120 °C	Atmospheric pressure	30 s–20 min	[118]
Ultrasound-assisted extraction	Water, aqueous and non-aqueous solvent	20–80 °C	Atmospheric pressure	10–60 min	[119,120]
Pulsed electric field extraction	Water, aqueous and non-aqueous solvent	20–50 °C	1.32–1.64 bar or atmospheric pressure	5 min–48 h	[121,122,123]
Enzyme-assisted extraction	Water, aqueous andnon-aqueous solvent	33–67 °C	Atmospheric pressure	20 min–4 h	[124,125]
Supercritical fluid extraction	Supercritical Fluids such as S-CO_2_, S-H_2_O	40–80 °C	35–70 MPa	10–60 min	[126,127]
High hydrostatic pressure extraction	Water, ethanol, glycerol, silicon oil, or a mixture of solvents	Below 45 °C	100–1000 MPa	3–15 min	[128,129,130]
Liquid gas extraction	Liquified petroleum gas (propane, n-butane), dimethyl ether	35 °C	Room temperature or low pressure 200–1000 kPa	20 min	[10,131]
Natural deep eutectic solvent extraction	Deep eutectic solvents such as reline, ethaline, glycerine, etc.	25–105 °C	Atmospheric pressure	30–60 min	[132,133,134]

**Table 3:** Polarity of various extraction solvents.

Solvent	Polarity Index	Relative Polarity
Water	9	1
Acetic acid	6.2	0.648
Ethanol	5.2	0.654
Methanol	5.1	0.762
Acetone	5.1	0.355
Ethyl acetate	4.4	0.228
Methyl acetate	4.4	0.253
Chloroform	4.1	0.259
Butanol	4	0.586
Isopropanol	3.9	0.546
Benzene	2.7	0.111
Toluene	2.4	0.099
Cyclohexane	0.2	0.006
Hexane	0	0.009

## Data Availability

Data are already present in the manuscript.
